# Mechanisms of isorhamnetin inhibition of osteoclast differentiation: insights from molecular dynamics simulations and *in vitro*/*in vivo* experiments

**DOI:** 10.3389/fphar.2025.1551257

**Published:** 2025-04-28

**Authors:** Yi Zhou, Shaoshuo Li, Bowen Hong, Zihan Wang, Yang Shao, Mao Wu, Jianwei Wang

**Affiliations:** ^1^ Graduate School, Nanjing University of Chinese Medicine, Nanjing, China; ^2^ Wuxi Affiliated Hospital of Nanjing University of Chinese Medicine, Wuxi, China

**Keywords:** isorhamnetin, molecular dynamics simulation, network pharmacology, osteoclast, osteoporosis

## Abstract

**Background:**

Osteoporosis (OP) represents a widespread bone remodeling disorder within the domain of orthopedics, markedly compromising the quality of life in the elderly population. The need to develop more efficient therapeutic approaches to attenuate bone resorption by suppressing the excessive activation of osteoclasts (OCs) remains urgent. The plant flavonoid Isorhamnetin (Iso), recognized for its potent antioxidant properties, has been the subject of extensive research regarding its potential in treating bone-related conditions.

**Method:**

This study adopts a comprehensive methodology to evaluate Iso’s impact on bone metabolism and its therapeutic possibilities for treating OP. By integrating network pharmacology, molecular dynamics simulations, and surface plasmon resonance (SPR), we performed *in vitro* phenotypic analyses to systematically evaluate the inhibitory effect of Iso on OC differentiation. The mechanisms behind Iso’s inhibition of OC differentiation were further elucidated. *In vivo* testing was also performed to substantiate the therapeutic effects of Iso in an OP animal model.

**Results:**

At low concentrations, Iso showed no cytotoxicity and did not interfere with cell proliferation in RAW 264.7 cells. Iso effectively inhibited RANKL-induced osteoclast differentiation in these cells, while downregulating related genes levels (*Nfatc1*, *Cts*k, *Trap*, *c-Fos*). Molecular dynamics simulations and surface plasmon resonance confirmed Iso’s dual binding to both RANKL and RANK. KEGG pathway enrichment analysis results indicated that Iso modulates the MAPK, NF-κB/PI3K-AKT, and calcium signaling pathways. Western blot analysis revealed that Iso treatment targeting the RANKL/RANK binding pathway significantly downregulated phosphorylation levels of JNK, P38, AKT, and p65. Concurrently, Iso stimulation markedly increased IκBα expression, thereby rescuing its degradation. Furthermore, Iso demonstrated a robust inhibitory effect on reactive oxygen species levels *in vitro*. Furthermore, in OVX mice, Iso treatment increased bone density, modulated serum bone metabolism markers, and downregulated transcriptional levels of OC marker genes.

**Conclusion:**

Iso exhibits therapeutic potential for OP by selectively targeting and disrupting the RANKL-RANK interaction. This intervention modulates the expression of intracellular transcription factors and multiple signaling pathways, thereby inhibiting the maturation of OCs. Through mitigating OC-mediated bone loss, Iso holds significant promise as a potent therapeutic agent for OP.

## 1 Introduction

Osteoporosis (OP) is a chronic, systemic skeletal disorder originating from a variety of etiologies, marked by the disruption of bone microarchitecture, a decline in bone density and quality, and the deterioration of the biomechanical properties of bone, ultimately resulting in pain, skeletal deformities, and even fractures (Compston et al., 2019). According to the International Osteoporosis Foundation, approximately 200 million individuals globally are affected by OP. Projections indicate that, by 2050, the number of hip fractures attributed to OP will rise from approximately 1.25 million cases in 1990 to 6.3 million cases worldwide (Arceo-Mendoza and Camacho, 2021). Consequently, identifying effective OP treatments is paramount to alleviating pain in elderly patients, improving survival rates, and enhancing the health-related quality of life.

The skeleton is a dynamic organ that preserves its function through a continuous remodeling process. This process involves bone formation, orchestrated by osteoblasts, and bone resorption, facilitated by osteoclasts (OCs), maintaining a delicate equilibrium (Liu et al., 2018). Bone remodeling sustains the mineralized component of bone, as OCs resorb aged bone, while osteoblasts replace the resorbed bone with new tissue by depositing collagen and minerals (Manolagas, 2000). Imbalances in skeletal resorption and formation processes may trigger numerous bone-related conditions, including Paget’s disease, rickets, OP, and others (de Villiers, 2024). Overactive OC function results in heightened bone degradation, compromising the skeletal system’s structural stability (Weitzmann and Ofotokun, 2016). Therefore, identifying agents capable of inhibiting osteoclastogenesis and bone resorption is of significant interest in preventing and treating pathological bone loss. Natural compounds represent a promising avenue for investigating potential therapies for postmenopausal OP (Zhang et al., 2022).

OCs originate from monocyte-macrophage precursors and play crucial roles in normal development and various disease states (Hasegawa and Ishii, 2022). OC formation involves an intricate system of cytokines and signal transduction mechanisms. Specifically, the Receptor Activator of Nuclear Factor-κB Ligand (RANKL) performs a vital regulatory role in OC formation, both experimentally and within living organisms (Du et al., 2020). When RANKL connects with receptor activator of NF-κB (RANK), it initiates the assembly of tumor necrosis factor receptor-associated factors (TRAFs), which then stimulate the mitogen-activated protein kinase (MAPK) and nuclear factor-κB (NF-κB) signal transduction pathways (Häcker et al., 2011). The activation of these mechanisms prompts the movement and subsequent stimulation of NFATc1, a fundamental transcriptional regulator in OC development (Takayanagi, 2021). NFATc1 specifically controls the production of numerous OC-related genes, encompassing TRAcP, CTR, and MMP-9 (Tokunaga et al., 2020). Therefore, targeting RANKL-induced signaling cascades to modulate NFATc1 expression offers significant therapeutic potential for treating OC-driven diseases, including postmenopausal OP.

Isorhamnetin (Iso, C_16_H_12_O_7_, CAS No. 480-19-3) is a key bioactive compound in *Hippophae rhamnoides L. (H. rhamnoides L.) and Ginkgo biloba (Ginkgo biloba L.).* It belongs to the flavonoid class and features an aromatic heterocyclic structure (Lei et al., 2024). Studies highlight its broad pharmacological potential, including cardiovascular protection, anti-inflammatory effects, and antioxidant activity (Gong et al., 2020). Notably, Iso shows promise as an anti-osteoporosis and anticancer agent. Its anticancer mechanisms include inducing cell cycle arrest, modulating apoptosis and autophagy, and suppressing cancer cell invasion and metastasis. Research demonstrates efficacy against breast, lung, prostate, and other cancers (Sager, 2023). Iso reduces breast cancer MDA-MB-231 cell invasion by downregulating MMP-2 and MMP-9. This effect involves suppression of P38/MAPK and STAT3 signaling (Li et al., 2015). Iso protects cardiovascular health by reducing inflammation and apoptosis. TNF-α, a key inflammatory cytokine, drives atherosclerosis development. Conversely, endothelial nitric oxide synthase (eNOS) preserves vascular function (Bae et al., 2022; Förstermann and Münzel, 2006). Research shows Iso counters TNF-α-induced endothelial injury. It blocks NF-κB and AP-1 signaling pathways, reduces adhesion molecule expression, and boosts eNOS activity (Nebigil and Désaubry, 2018). Inflammation drives many diseases through interactions between inflammatory cells, signaling factors, and molecular pathways. NF-κB, a key transcription factor, controls the expression of inflammatory genes (Sun et al., 2022). Iso fights inflammation by blocking the JNK and AKT/IKK pathways linked to NF-κB activation. This mechanism suggests Iso could treat acute inflammatory disorders (Yang et al., 2013). Iso also reduces TNF-α-induced inflammation in bronchial epithelial cells. It improves asthma-related airway inflammation and tissue remodeling by suppressing MAPK and NF-κB signaling (Ren X. et al., 2021). Several studies have also been conducted on the treatment of OP. These studies indicate that Iso can markedly enhance osteoblasts’ differentiation, mineralization, migration, and adhesion. Its mechanism of action is believed to operate through activating the ERK-dependent BMP2-Smad signaling pathway, thereby conferring protective effects in OP (Li et al., 2024). Furthermore, Iso has been shown to inhibit OC formation and activity by suppressing reactive oxygen species production and downregulating osteoclastogenesis-related genes and signaling pathways, encompassing MAPK, NF-κB, and AKT. In a murine model of osteoarthritis, Iso treatment reduced excessive OC activity and cartilage damage, underscoring its potential as a therapeutic agent targeting OC function in osteoarthritis (Zhou et al., 2019). Currently, investigations have not yet examined Iso’s direct influence on OCs in the context of OP. Consequently, this study is focused on investigating how Iso can reduce bone resorption by inhibiting OC function, thereby improving bone density and enhancing bone structure. Using an animal model specific to OP, this research combines traditional *in vitro* experiments with modern bioinformatics techniques. By employing complementary validation approaches, the suppressive impact of Iso on RANKL-mediated OC development was extensively examined. The study seeks to establish a solid scientific basis for the prospective therapeutic use of Iso in managing OP.

## 2 Materials and methods

### 2.1 Preparation of iso and cell viability and proliferation assay

Iso was procured from Chamface Biological Company (CFN98735, China). It was solubilized in dimethyl sulfoxide (DMSO) and kept at −80°C for subsequent usage. Mouse Mononuclear Macrophages Cells (RAW 264.7) was procured from Procell and maintained in α-MEM (SH30265.01, Hyclone, USA) comprising 10% fetal bovine serum (FBS) (10099141C, Gibco, USA). Experiments were conducted using cells between passages 8 and 10. RAW 264.7 cells were seeded into 96-well plates at 3 × 10^3^ cells/well once they reached about 90% confluence. After a 24-h incubation period, the cells were exposed to Iso at varying concentrations (0, 1.25, 2.5, 5, 10, and 20 μM). Following a 48-h cultivation at 37°C in a moisture-saturated environment with 5% CO_2_, 10 μL of Cell Counting Kit-8 (CCK-8) solution (C0037, Beyotime, China) was introduced to each well. The plates were kept for an additional 2 h in the dark. Absorbance was then measured at 490 nm utilizing a microplate reader, and cell viability was denoted as a percentage relative to untreated controls.

### 2.2 Tartrate-resistant acid phosphatase (TRAP) staining

#### 2.2.1 Iso attenuates RANKL-induced osteoclastogenesis

RAW 264.7 cells were initially differentiated and subsequently placed into 96-well cell culture plates at an optimal density. After 24 h, the culture medium was substituted with complete α-MEM comprising 50 ng/mL receptor activator of nuclear factor kappa-Β ligand (RANKL) (RD-462-TEC-010, RD), 10% FBS, and 1% Penicillin-Streptomycin, along with varying concentrations of Iso (2.5, 5, 10, and 20 μM). The medium was refreshed every 48 h for 6–7 days until mature osteoclasts were formed. The cells underwent fixation utilizing 4% paraformaldehyde for 15 min at ambient conditions and were processed with a TRAcP staining kit (SLBZ2669, Sigma, Germany) per the supplier’s protocol. After staining, 100 μL of TRAP staining solution was introduced to each well. The stained cells were subsequently imaged with an inverted microscope, and the number of TRAP-positive multinucleated cells (≥3 nuclei) per well was quantified using ImageJ software.

#### 2.2.2 Time-course analysis of iso effects on OC differentiation

RAW 264.7 cells were placed into 96-well plates at 3 × 10^3^ cells/well. On days 1, 3, and 5, the culture medium was substituted with complete α-MEM comprising either 0 or 20 μM Iso. The medium was refreshed every 48 h for 6–7 days until mature osteoclasts were emerged. Subsequently, the cells underwent fixation, staining, and analysis procedures, as depicted in Section 2.2.1.

### 2.3 F-actin staining

RAW 264.7 cells were seeded in 96-well plates and treated with 50 ng/mL receptor activator of nuclear factor-kappa-B ligand (RANKL) alongside Iso (0, 2.5, or 20 μM). A negative control group, without RANKL induction, was established for comparison. The medium was refreshed every 48 h for 6–7 days until mature osteoclasts had developed. Subsequently, the cells underwent fixation with 4% paraformaldehyde at ambient temperature for 10 min, then permeabilization utilizing 0.5% Triton X-100 solution for 10 min. The samples were rinsed three times with PBS before being exposed to phalloidin solution under dark conditions for 1 h to visualize F-actin. The cell nuclei were then labeled with DAPI for 10 min. A Nikon confocal microscope was employed to capture fluorescent images, and ImageJ software was utilized to analyze the actin ring areas and perform nuclear counting.

### 2.4 Generation of intracellular ROS

RAW 264.7 cells were seeded in 96-well plates and treated with 50 ng/mL receptor activator of nuclear factor-kappa-B ligand (RANKL) alongside Iso (0, 2.5, or 20 μM). A negative control group, without RANKL induction, was established for comparison. After 60 min of treatment, incubate RAW 264.7 cells in Hank’s balanced salt solution (HBSS) containing 10 µM DCFH-DA (S0034, Beyotime) for 20 min at 37°C. Capture fluorescent images using a Nikon confocal microscope. Quantify actin ring areas and nuclei counts using ImageJ software.

### 2.5 Immunofluorescence for the detection of P65

Seed RAW 264.7 cells in a 96-well plate. Treat with 50 ng/mL RANKL and Iso (0, 2.5, or 20 μM). A negative control group, without RANKL induction, was established for comparison. Following permeabilization using 0.25% Triton X-100 in PBS, nonspecific antibody interactions were reduced by incubating the cells with blocking agents such as serum. Incubate cells overnight with anti-P65 primary antibody (diluted in blocking buffer). Wash cells. Add fluorophore-conjugated secondary antibody. Incubate for 2 h. Counterstain nuclei with DAPI (1 μg/mL). Mount coverslips and image using a Nikon confocal microscope.

### 2.6 Real-time quantitative polymerase chain reaction (RT-qPCR)

Following the seeding of cells into a 6-well culture plate and their adhesion, a complete α-MEM medium supplemented with receptor activator of nuclear factor kappa-B ligand (RANKL) (50 ng/mL) was introduced alongside Iso at distinct concentrations (0 μM, 10 μM, and 20 μM). The Cell Total mRNA Isolation Kit (19221ES50, Yeasen, China) was utilized to obtain total mRNA, and a reverse transcription kit (R323-01, Vazyme, China) enabled the synthesis of cDNA from 1 µg mRNA. Gene expression analysis was conducted using 20 µL SYBR qPCR Master Mix (11202ES08, Yeasen, China) on a fluorescent RT-qPCR system (Applied Biosystems 7500, USA), with GAPDH serving as the housekeeping gene. The 2^-△△Ct^ methodology was applied for gene expression calculations, with results expressed as fold changes compared to the reference gene. Each experiment was performed in triplicate. The specific information of primer sequences is shown in Table 1.

**TABLE 1:** Primers for RT-qPCR.

Genes	Forward (5–3)	Reverse (5–3)	Tm (°C)
*β-actin*	GAG​AGG​GAA​ATC​GTG​CGT​GAC​ATC​A	ATC​GGA​ACC​GCT​CGT​TGC​CAA​TAG​T	54
*Nfatc1*	TCT​CAC​CAC​AGG​GCT​CAC​T	TTT​CTG​GAA​GCA​ACG​GGA​T	56.1
*Ctsk*	GAACGAGAAAGCCCTGAA	AGCCCACCACCAACACT	51.5
*c-Fos*	CCGAAGGGAACGGAATA	TGGGAAGCCAAGGTCAT	53
*Mmp9*	CTC​AAG​TGG​GAC​CAT​CAT​AAC​A	GATACCCGTCTCCGTGCT	55.4

### 2.7 Western blot

RAW 264.7 cells were plated into 6-well culture plates and allowed to adhere. Osteoclast differentiation was induced by supplementing complete α-MEM with receptor activator of nuclear factor kappa-B ligand (RANKL) (50 ng/mL) and Iso (0 or 20 μM). We assessed the concentration-dependent effects of Iso on osteoclast-associated protein expression. For consistency, 20 μM Iso was administered at matched intervals (days 1, 3, 5). An acute protocol involving timed interventions delivered at 0, 10, 20, 30, and 60-min intervals was implemented. Protein was procured from the cells utilizing 200 µL of RIPA lysis buffer per well, comprising phosphatase inhibitor A and a protease inhibitor cocktail. After a 30-min incubation on ice, protein levels were determined via BCA assay. Protein separation was accomplished through SDS-PAGE before membrane transfer. Primary antibody incubation occurred overnight at 4°C, while secondary antibodies were applied for 1 h at ambient temperature. Protein detection utilized high-sensitivity ECL chemiluminescence methodology. A Bio-Rad imaging system enabled protein visualization, with Image software facilitating quantification. Antibodies employed for West blot analysis included those specific to ERK, JNK, P38, P65, IκB-α, PI3K, AKT, CTSK, MMP9, c-Fos, NFATc1, CaMK4, CaMK2, and Calmodulin1/2/3, with GAPDH serving as the loading control. Detailed information regarding the antibodies used can be found in Supplementary Table S1.

### 2.8 Network pharmacology analysis

Target information and structure for Iso were obtained from PubChem (CAS: 480-19-3) and saved in PDB format for further analysis. Target prediction was performed using the SwissTargetPrediction database with the PDB file of Iso (Daina et al., 2019). Genes associated with osteoporosis were retrieved from the GeneCards database (Version 5.23) by searching for “osteoporosis”. The therapeutic targets of Iso in the context of osteoporosis were analyzed using Venny software. Enrichment analyses for Kyoto Encyclopedia of Genes and Genomes (KEGG) pathways and Gene Ontology (GO) terms of differentially expressed genes were conducted using the ClusterProfiler package (version 3.18.0) in R. Pathways with parameters set as *p*. *adjust* <0.05 and *q-value* <0.025 were considered significantly enriched. (Feng et al., 2024; Miliotis et al., 2024). In the enrichment analysis, different colors represent different significance levels, with purple indicating smaller *q-values*. The size of each circle is proportional to the number of enriched genes. The 2D structure of Iso was converted to MOL2 format using CHEM 3D as a small molecule ligand. Target protein structures were procured from the PDB database and processed using PyMOL software (version 2.3.6) to remove water molecules and identify the original ligands from the core target proteins. Processed protein targets were imported into AutoDock software (version 4.2.0) for charge calculation and atom type assignment. Molecular docking was carried out using AutoDock-Vina software (v1.1.2) to assess the compound-ligand complex affinity and generate comprehensive docking scores. Visualization of the molecular docking results was performed using Discovery Studio, and the effectiveness of docking was evaluated based on affinity values.

### 2.9 Molecular dynamics simulation

Molecular dynamics simulations were executed utilizing the GROMACS software package to explore the interactions between small molecule compounds and target protein molecules (Van Der Spoel et al., 2005; Abraham et al., 2015). Structure files for both protein and small molecules were separately imported into GROMACS. Topology files and simulation boxes were produced utilizing the pdb2gmx and gmx editconf commands, respectively. Prior to the simulation, energy minimization was performed on both protein and small molecule structures.

A 100 ns molecular dynamics simulation was executed on the protein-compound complexes using the gmx grompp and gmx mdrun commands. Conformational changes were monitored throughout the simulation. Post-simulation analysis was conducted with the gmx rms command to calculate several parameters, including root-mean-square deviation (RMSD), root-mean-square fluctuation (RMSF), radius of gyration (Rg), solvent-accessible surface area (SASA), and hydrogen bonding interactions (Hbind) between the proteins and small molecule compounds.

The results were analyzed and visualized through graphical representations and statistical tables. To further assess the accuracy of the protein-compound docking results, the Molecular Mechanics Poisson-Boltzmann Surface Area (MM/PBSA) method was utilized. This approach calculates the binding free energies between the proteins and small molecule compounds, thereby comprehensively evaluating the docking results. By calculating the complex’s total free energy, the stability of the protein-compound interactions can be determined, complementing the structural and dynamic insights obtained from the molecular dynamics simulations.

### 2.10 Surface plasmon resonance (SPR) analysiss

All SPR experiments were performed using a Biacore t200 instrument (Cytiva, Sweden). The CM5 series sensor chip (Cytiva BR100012) was activated with a mixture of NHS/EDC (100 μL each, 10 μL/min flow rate, 600 s activation) from the Amine Coupling Kit (Cytiva BR100050). RANK protein and RANKL protein (30–50 μg/mL in Acetate 4.0 buffer, Cytiva BR100349) were immobilized at a flow rate of 10 μL/min for 600 s, followed by a 420 s blocking step with Ethanolamine. Iso, serially diluted in PBST buffer (0.01% Tween20/PBS, filtered through 0.22 μm) containing 5% DMSO and 0.1% BSA, were injected at 30 μL/min in HBS-EP+ running buffer (Cytiva BR100669) at 25°C, with 60 s association and 180 s dissociation phases. Reference flow cell subtraction was applied for background correction. The experiment utilized a dual-channel mode (2-1/4-3).

### 2.11 *In vivo* experiments

Female ICR mice (9 weeks old) were procured from the Jiangnan University Experimental Animal Center and housed in a controlled environment with a 12-h light/dark cycle, humidity maintained between 55% and 60%, and temperatures ranging from 22°C to 24°C. *Ad libitum* access to food and water was provided to the mice. All experimental procedures were conducted per the ethical guidelines established by the Wuxi Traditional Chinese Medicine Hospital Ethics Committee (Ethics Approval No. SWJW2023030301). After 1 week, at 10 weeks of age, 36 mice were randomly assigned to six groups, with six mice in each group. The groups included: a sham surgery group, an OVX group, and three OVX + Iso groups with doses of 10 mg/kg, 20 mg/kg, and 40 mg/kg, respectively.

Relevant literature on drug intervention dosages and modeling techniques was reviewed (Zhou et al., 2019; Deng et al., 2023). The dorsal region was selected as the entry site, and the mice were anesthetized using isoflurane. After proper sterilization, bilateral ovariectomy was performed to establish the OVX model. In the sham surgery group, the ovaries were exposed without removal and subsequently repositioned to their original location. Following a one-week postoperative recovery period, the mice were given standard food and water. The OVX model was considered fully established approximately 8 weeks after surgery. Before initiating formal testing, preliminary dose-ranging studies were performed to identify Iso’s effective dosage. Through systematic evaluation of escalating doses (10–40 mg/kg), 40 mg/kg emerged as the optimal concentration, demonstrating significant suppression of bone resorption activity. Drug interventions commenced in the 9th week post-surgery. Mice in the Iso treatment groups received intraperitoneal injections of Iso at the specified concentrations every other day. Mice in the OVX and sham surgery groups were administered intraperitoneal injections of equal volumes of DMSO and physiological saline every other day. The intervention period lasted for a total of 8 weeks. To maintain blinding, both surgical and pharmacological personnel were unaware of group assignments.

Two hours after administering the final dose, the mice were anesthetized with isoflurane. Once anesthesia was properly induced, the bilateral whiskers of the mice were shaved, and blood samples were collected by enucleating the eyeball and transferring the blood into microcapillary tubes. The collected blood was allowed to stand at 4°C for 2 h, followed by centrifugation at 4°C at 2,500 rpm for 10 min to isolate the serum. The serum was then transferred to cryovials and stored at −80°C for subsequent analysis of bone metabolism markers via enzyme-linked ImmunoSorbent assay (ELISA). After blood collection, the mice were euthanized, and their bilateral femurs and tibias were harvested. The left femur from each group was placed into a cryovial containing 4% paraformaldehyde and stored at room temperature for Micro-CT scanning and histological staining of bone tissue. The tibias were placed into 2 mL cryovials, rapidly frozen in liquid nitrogen, and stored at −80°C for quantitative PCR analysis. Statistical analyses were performed by a third-party researcher who was not involved in the experimental procedures and remained blinded to group allocation.

#### 2.11.1 Micro-CT scanning

The proximal tibia was evaluated through high-resolution Micro-CT imaging following the intervention. The right tibia of each mouse was carefully excised, preserved in 4% paraformaldehyde (PFA) for 2 days, and then transferred to 75% alcohol for long-term storage (SkyScan 1176, Bruker). The Micro-CT scanning parameters were set as follows: current of 382 μA, voltage of 65 kV, with a 0.5 mm AI filter, and a pixel size of 4000 × 2672 (9 µm). The proximal tibia was subsequently scanned and analyzed utilizing CTAn and CTVox software, and 3D reconstructions were generated to evaluate trabecular number (Tb.N), bone mineral density (BMD), bone volume/tissue volume (BV/TV), trabecular thickness (Tb.Th), and trabecular separation (Tb.Sp).

#### 2.11.2 Histological analysis

The tibias were subjected to decalcification for 21 days in a 10% Ethylenediaminetetraacetic acid (EDTA) solution following the Micro-CT scan. Upon completion of decalcification, the tibias were embedded in paraffin and subsequently sectioned into 5 µm-thick slices. These sections were then stained using hematoxylin and eosin (H&E) and subjected to tartrate resistant acid phosphatase (TRAP) activity staining.

#### 2.11.3 ELISA

ELISA was utilized to assess serum markers related to bone metabolism in mice, including markers of bone resorption, such as TRAP (ml063508, mlbio), type I collagen C-terminal peptide (β-CTX) (ml237021, mlbio), and collagen pyridine cross-linking (PYD) (ml390411, mlbio). The procedures were strictly adhered to as outlined in the reagent kit instructions.

#### 2.11.4 RT-qPCR

The tibias from each group of mice, previously stored at −80°C, were retrieved, and the bone tissue was pulverized using bone forceps. The fragments were subsequently transferred into a pre-warmed mortar (heated at 60°C for 2 h). An adequate volume of liquid nitrogen was then introduced into the mortar, and the bone fragments were rapidly ground. Efforts were made to replenish the liquid nitrogen as it evaporated, ensuring it remained in contact with the bone fragments. The grinding procedure was repeated until the bone fragments were reduced to a fine powder. Total RNA extraction, reverse transcription, and fluorescence quantitative PCR analysis were carried out as outlined in section “2.6”. Detailed information regarding the primers used can be found in Supplementary Table S2.

### 2.12 Data analysis

All experiments were conducted in triplicate, adhering to established pharmacological experimental design protocols. The data are presented as mean ± standard deviation (SD). Statistical analysis was performed using either one-way or multifactor analysis of variance (ANOVA), with a *p*-*value* below 0.05 deemed statistically significant.

## 3 Results

### 3.1 Iso inhibits OC differentiation

**FIGURE 1:**
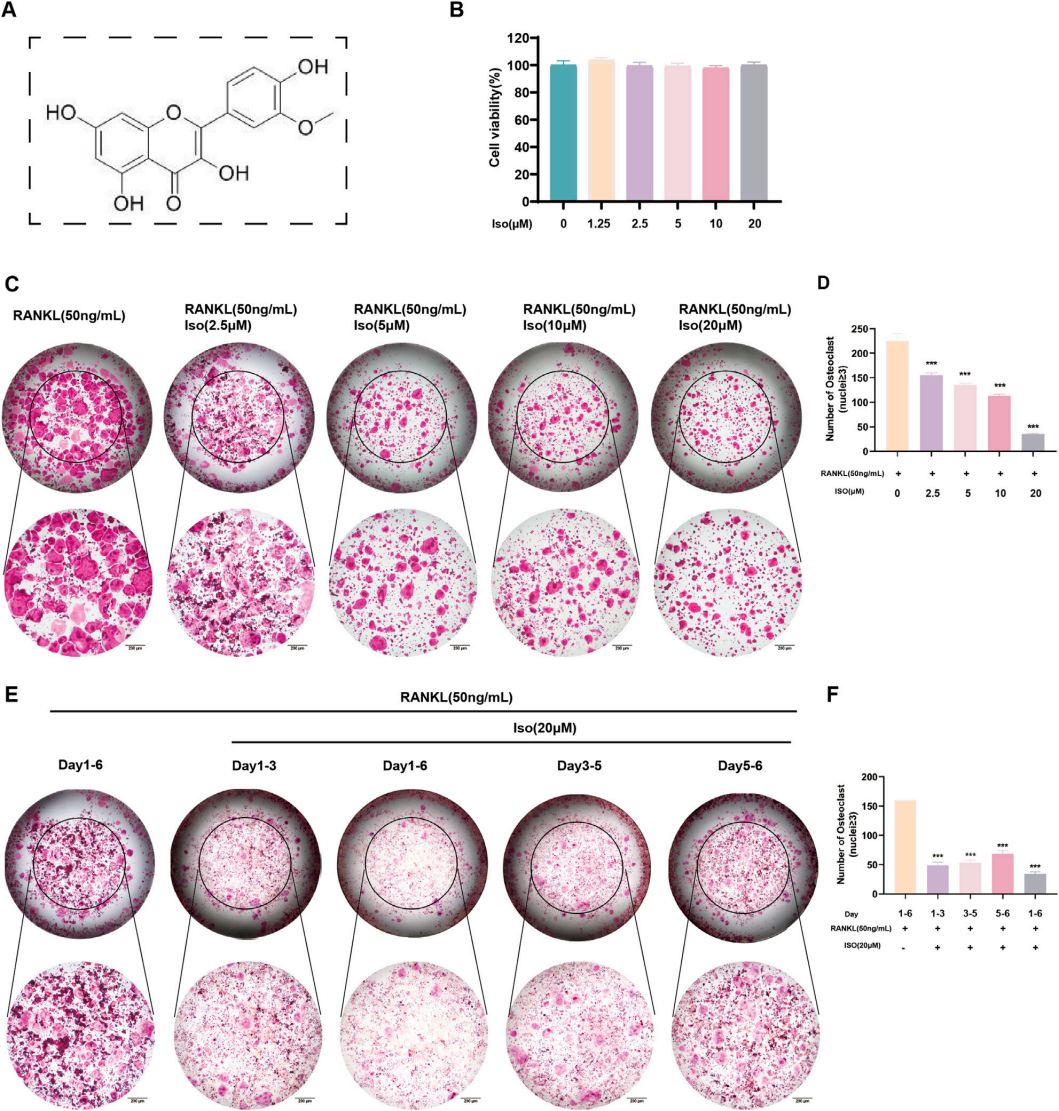
Iso inhibits osteoclast formation. **(A)** Chemical structure of Iso. **(B)** Iso at concentrations ≤20 μM showed no significant effects on RAW 264.7 cell proliferation or viability. **(C,D)** TRAP staining was employed to evaluate the dose-dependent effects of Iso (0, 2.5, 5, 10, and 20 μM) on osteoclast differentiation, with quantitative assessments confirming the observed trends. **(E,F)** To evaluate the effect of Iso on osteoclast maturation, TRAP staining and quantitative analysis were performed at multiple time points. Iso: Isorhamnetin. *p < 0.05, **p < 0.01, ***p < 0.001 compared to the control group (n = 3). Scale bars = 200 μm.

The inhibitory effects of Iso on osteoclast differentiation and formation were evaluated through TRAP staining. The chemical structure of Iso is presented in Figure 1A, while the outcomes of the 48-h CCK-8 assay are shown in Figure 1B. The results demonstrated that Iso concentrations up to 20 μM did not exhibit cytotoxicity nor influence RAW 264.7 cell proliferation. We selected the highest non-toxic concentration of Iso (20 μM) for subsequent cell experiments. TRAP staining showed that Iso reduced the formation of TRAP-positive multinucleated osteoclasts in a concentration-dependent manner. These osteoclasts contained three or more nuclei per cell (Figures 1C,D). To identify the specific phase of osteoclastogenesis influenced by Iso, 20 μM (the maximal inhibitory dose) was introduced into RANKL-supplemented RAW 264.7 culture medium during predefined incubation intervals. Our data show Iso exerts stronger suppression on TRAP-positive osteoclast numbers during early differentiation (Days 1–3) than in later phases (Figures 1E,F). This indicates that Iso’s anti-osteoclastogenic action targets early differentiation processes. Together, these results show that Iso reduces osteoclast maturation in a dose-dependent manner. Its strongest effects occur during the early stages of osteoclast development.

### 3.2 Iso inhibited the formation of F-actin in osteoclasts

**FIGURE 2:**
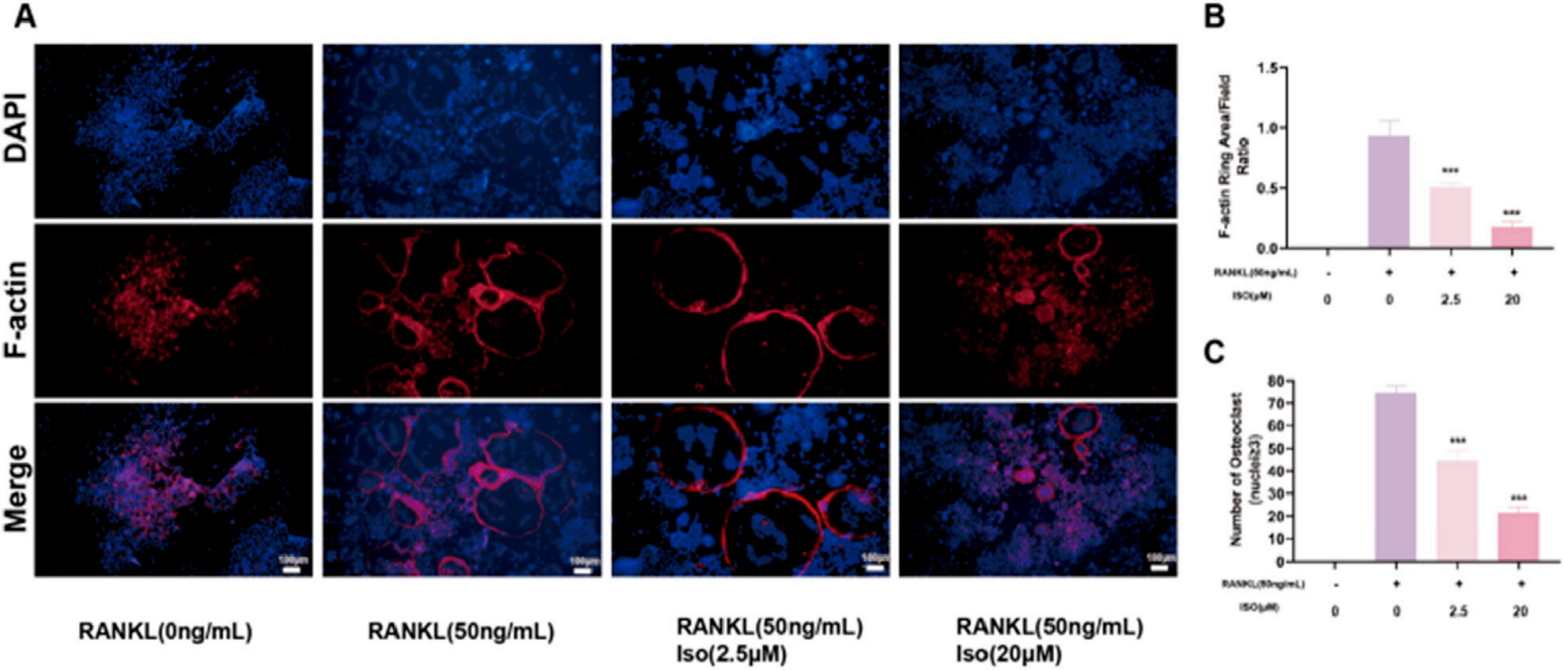
Iso attenuated the development of osteoclast-specific podosome belt structures, a cytoskeletal hallmark critical for bone-resorptive function, as evidenced by diminished staining intensity. **(A)** Iso inhibits the cytoskeleton formation activity of mature osteoclast. **(B,C)** Quantitative analysis of osteoclast numbers is presented as bar graphs. Iso: Isorhamnetin. *p < 0.05, **p < 0.01, ***p < 0.001 compared to the control group (n = 3). Scale bars = 100 μm.

F-actin rings serve as hallmark indicators of osteoclast maturation, prompting our investigation into Iso’s influence on their development. For this purpose, low (2.5 μM) and high (20 μM) doses of Iso were chosen for evaluation. Post-induction treatment, rhodamine-labeled phalloidin staining was employed. Consistent with TRAP staining outcomes, Iso demonstrated a dose-dependent suppression of F-actin ring formation (Figure 2A). Additionally, statistical evaluations were conducted to quantify both the frequency of F-actin rings and the extent of F-actin coverage within osteoclasts (Figures 2B,C).

### 3.3 Iso restrains RANKL-induced ROS generation

**FIGURE 3:**
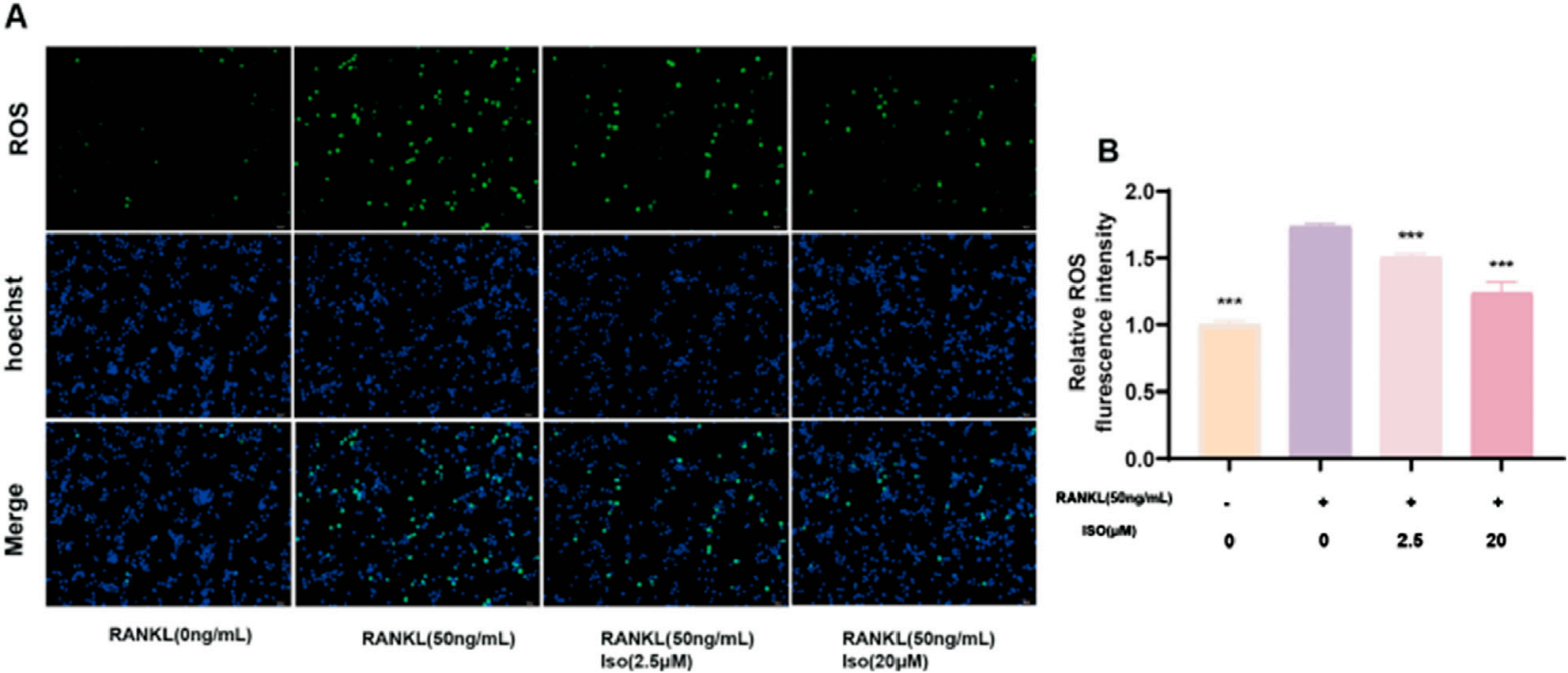
Iso Regulation of ROS Levels. **(A)** Representative fluorescence images depicting ROS levels in RAW 264.7 cells, with or without Iso treatment. **(B)** Quantification of ROS-positive cells in each representative field of view. Iso: Isorhamnetin. *p < 0.05, **p < 0.01, ***p < 0.001 compared to the RANKL (50 ng/mL) group (n = 3). Scale bars = 50 μm.

We measured intracellular ROS levels using the fluorescent probe DCFH-DA and fluorescence microscopy. This assessed Iso’s effect on RANKL-induced ROS production during osteoclast differentiation. In RAW 264.7 cells treated with RANKL and Iso (2.5 or 20 μM), DCF fluorescence intensity decreased dose-dependently (Figure 3A). Iso also significantly reduced ROS levels in these cells (Figure 3B). These findings indicate that Iso inhibits RANKL-driven osteoclast differentiation by suppressing ROS generation.

### 3.4 Iso inhibits expression of osteoclast differentiation-related genes and proteins

**FIGURE 4:**
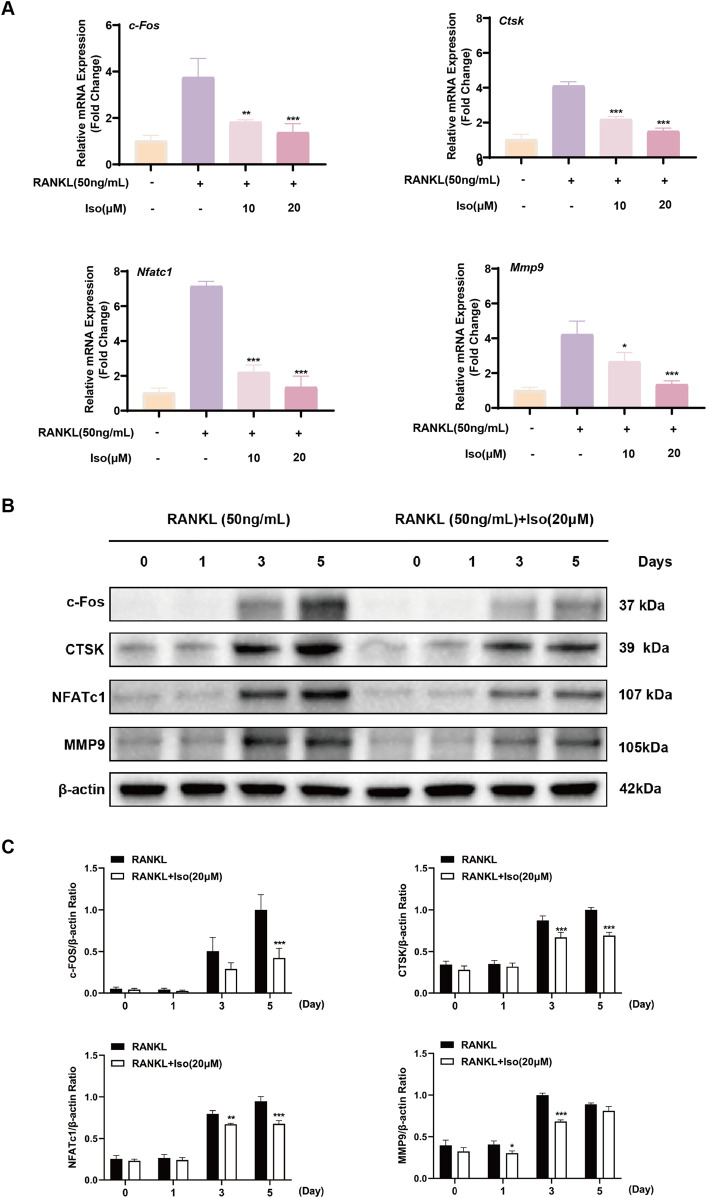
Iso inhibits the expression of osteoclast-related genes and proteins. **(A)** Dose-dependent inhibition of osteoclast-related gene expression by Iso. **(B,C)** Iso markedly inhibits the relative expression levels of CTSK, c-Fos, MMP9, and NFATc1 proteins. Iso: Isorhamnetin. *p < 0.05, **p < 0.01, ***p < 0.001 compared to the control group (n = 3).

NFATc1 and c-Fos are pivotal regulators of osteoclast differentiation, maturation, and functional activation. RANKL-TRAF6 binding initiates signaling cascades that activate transcription factors such as NF-κB and MAPK pathways (Han and Kim, 2019). Subsequent nuclear translocation of activated NFATc1 enables osteoclasts to execute bone-resorptive functions. To investigate these mechanisms, we used RT-qPCR and West blot to assess RANKL-driven osteoclast differentiation and Iso’s modulatory effects on this process. As illustrated in Figures 4A–C, the expression levels of c-Fos, CTSK, MMP9, and NFATc1 were suppressed by Iso at both the gene and protein levels during osteoclast differentiation. The inhibitory effects were enhanced as Iso concentrations increased. These effects were most pronounced on days 3 and 5 of differentiation, particularly for the proteins c-Fos, CTSK, MMP9, and NFATc1. Collectively, our findings validate Iso’s pharmacological activity through multifaceted approaches, revealing its capacity to suppress osteoclastogenesis and functional maturation in a dose-dependent manner. Nevertheless, the precise molecular targets and mechanisms underlying these effects remain incompletely characterized, necessitating additional studies to elucidate Iso’s mode of action.

### 3.5 Network pharmacology analysis results

**FIGURE 5:**
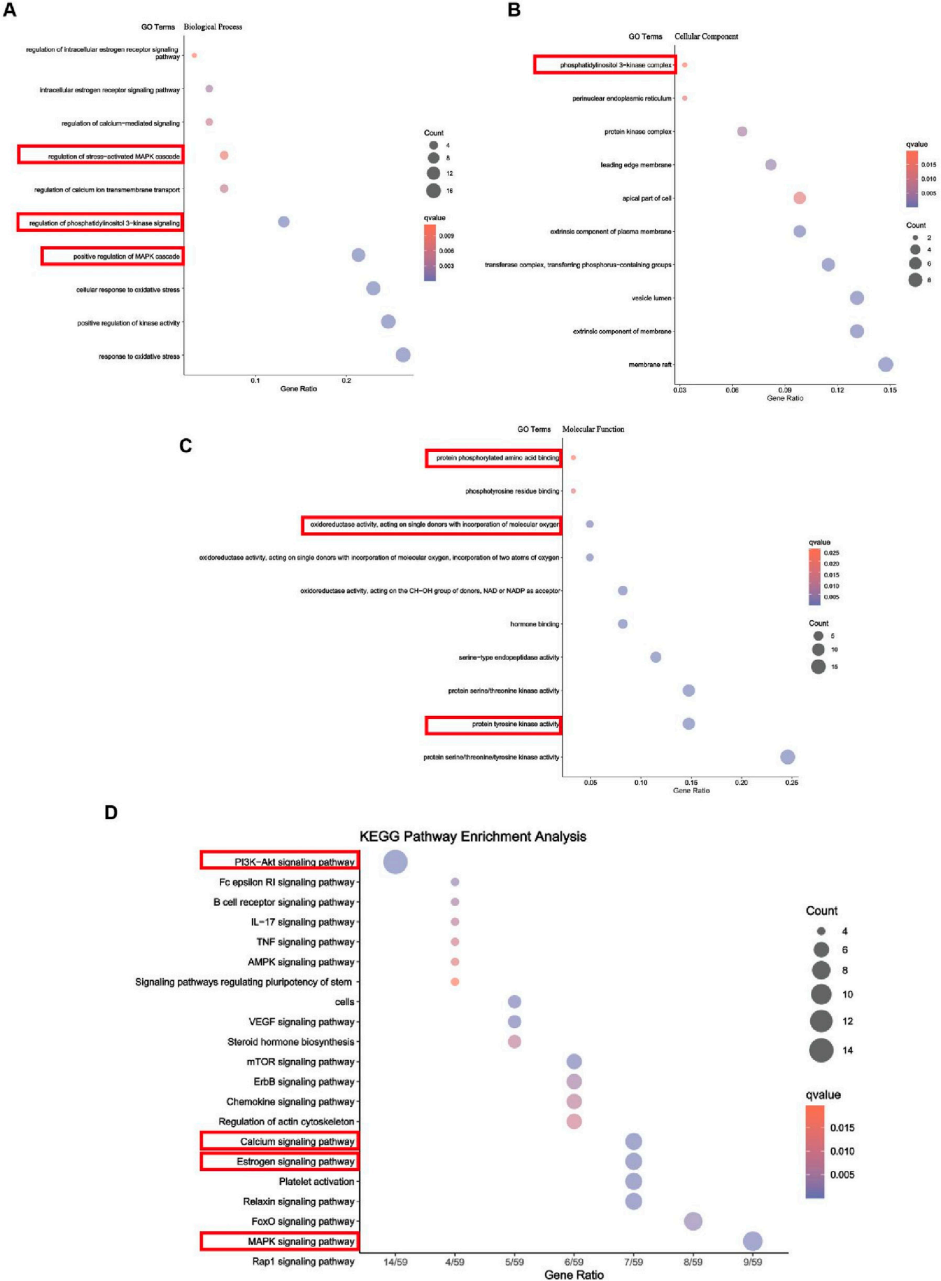
Function enrichment analysis. **(A)** Gene Ontology (GO) enrichment analysis (Biological Process). **(B)** GO enrichment analysis (Cellular Component). **(C)** GO enrichment analysis (Molecular Function). **(D)** Kyoto Encyclopedia of Genes and Genomes (KEGG) pathway enrichment analysis.

**FIGURE 6:**
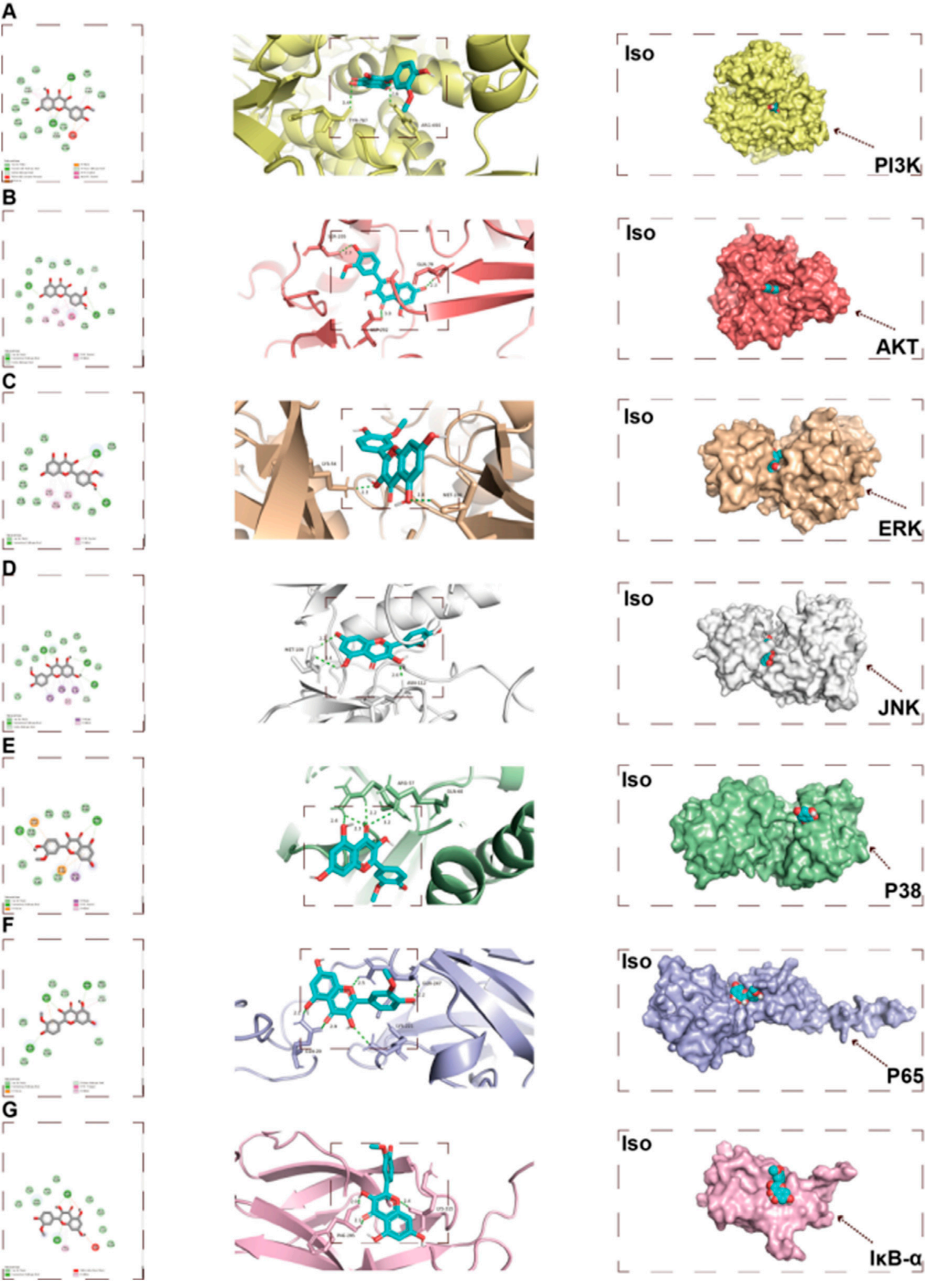
Molecular docking results. **(A–G)** Two-dimensional (2D) and three-dimensional (3D) schematic representations of Iso docking with target proteins. Iso: Isorhamnetin.

We employed network pharmacology to predict Iso’s potential therapeutic mechanisms against osteoporosis (OP). Subsequently, functional enrichment analysis was performed on the identified targets to elucidate its mode of action in OP management. Gene Ontology Biological Process (GO BP) classification analysis suggested that Iso may inhibit osteoclast differentiation via multiple signaling pathways, including the activation of the MAPK cascade, modulation of intracellular estrogen receptor signaling, and regulation of the stress-activated MAPK cascade (Figure 5A). In the Cellular Component (CC) category, Iso’s suppression of osteoclast differentiation was primarily associated with the protein kinase complex and the phosphatidylinositol 3-kinase (PI3K) complex (Figure 5B). In the Molecular Function (MF) category, the inhibition of osteoclast differentiation was primarily attributed to protein phosphorylated amino acid binding, oxidoreductase activity, acting on single donors with incorporation of molecular oxygen, and protein tyrosine kinase activity (Figure 5C). Moreover, the Kyoto Encyclopedia of Genes and Genomes (KEGG) pathway analysis revealed that Iso suppresses osteoclast differentiation through the MAPK, PI3K-AKT, and calcium ion signaling pathways (Figure 5D). To investigate Iso’s interaction with these pathways, we employed molecular docking simulations to construct a predictive binding model (Figure 6). Key targets within the pathways were prioritized, and binding interactions between Iso and these proteins were analyzed. Computational docking revealed that Iso exhibits strong binding affinities with PI3K (−8.2 kcal/mol), AKT (−9.9 kcal/mol), ERK (−7.8 kcal/mol), JNK (−8.2 kcal/mol), P38 (−8.8 kcal/mol), p65 (−7.2 kcal/mol), and IκB-α (−6.3 kcal/mol) (Figures 6A–G). Structural interaction details, including binding site residues, are summarized in Table 2. Table 2 illustrates that Iso demonstrated a significant binding affinity for ERK, JNK, P38, IκB-α, P65, PI3K, and AKT proteins, with the highest affinity observed for AKT and P38. Figure 6 depicts the 2D and 3D docking structures of Iso interacting with these proteins. Moving forward, our mechanistic investigations will prioritize the MAPK, PI3K-AKT, and calcium signaling cascades, with subsequent experimental validation of these pathway-associated targets.

**TABLE 2:** Docking affinity between Iso and protein.

Ligand	Receptor	Affinity (kcal/mol)	Binding sites
Iso	PI3K	−8.2	PHE: 694, ARG: 690, ARG: 849, TYR: 867
Iso	AKT	−9.9	GLN: 79, SER: 205, LEU: 264, VAL: 270, TRP: 80, LYS: 268
Iso	ERK	−7.8	ALA: 52, LEU: 156, VAL: 39, TYR: 36, ASP: 167
Iso	JNK	−8.2	ASN: 112, MET: 109, GLU: 107, VAL: 38, VAL: 156, ILE: 30, ALA: 51
Iso	P38	−8.8	GLU: 71, ARG: 57, HIS: 64, ALA: 34
Iso	P65	−7.2	GLN: 247, LYS: 221, GLN: 29, HIS: 181
Iso	IκB-α	−6.3	PHE: 295, LYS: 315, PRO: 314

### 3.6 Iso inhibits protein expression in the MAPK pathway

**FIGURE 7:**
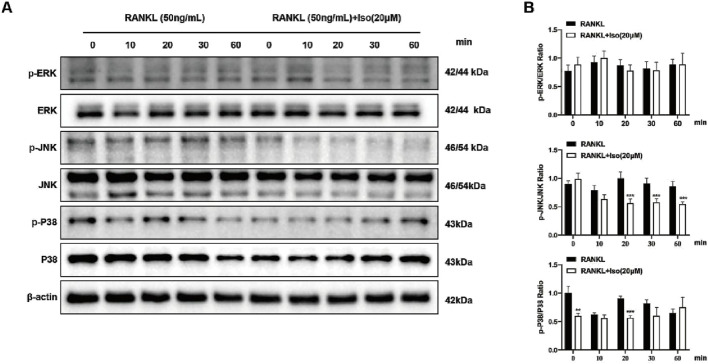
Iso inhibits osteoclastogenesis via the MAPK signaling pathway. **(A)** Representative Western blot analysis demonstrates Iso’s effects on RANKL-induced phosphorylation of ERK, JNK, and P38 MAPK signaling molecules. **(B)** Quantification of p-ERK, p-JNK, and p-P38 band intensity ratios relative to ERK, JNK, and P38 were analyzed (n = 3). Iso: Isorhamnetin. *p < 0.05, **p < 0.01, ***p < 0.001 compared to the control group (n = 3).

RANKL promotes the auto-amplification of NFATc1, a critical transcription factor for osteoclast differentiation, by triggering the phosphorylation of key MAPKs (ERK, JNK, P38). To elucidate the inhibitory mechanism of Iso on osteoclastogenesis and complement findings from network pharmacology and molecular docking analyses, this study was designed. The results of westbolt demonstrated that treatment with Iso (20 μM) markedly reduced JNK phosphorylation at various time points (20, 30, and 60 min). In contrast, the inhibition of P38 phosphorylation was transient, showing a notable reduction only at 20 min post-treatment, with no persistent suppression observed at 30 and 60 min (Figures 7A,B). Moreover, no significant changes in ERK phosphorylation levels were detected at any of the time points following Iso treatment (Figures 7A,B). Collectively, our research uncovered critical insights into the RANKL-regulated MAPK signaling cascade, demonstrating that Iso selectively suppresses phospho-JNK and phospho-P38 expression, with pronounced inhibitory effects on JNK activity. Leveraging these observations, we explored the therapeutic potential of Iso in modulating osteoclast differentiation and identified novel mechanistic pathways.

### 3.7 Iso inhibits osteoclastogenesis via NF-κB/PI3K-AKT pathway

**FIGURE 8:**
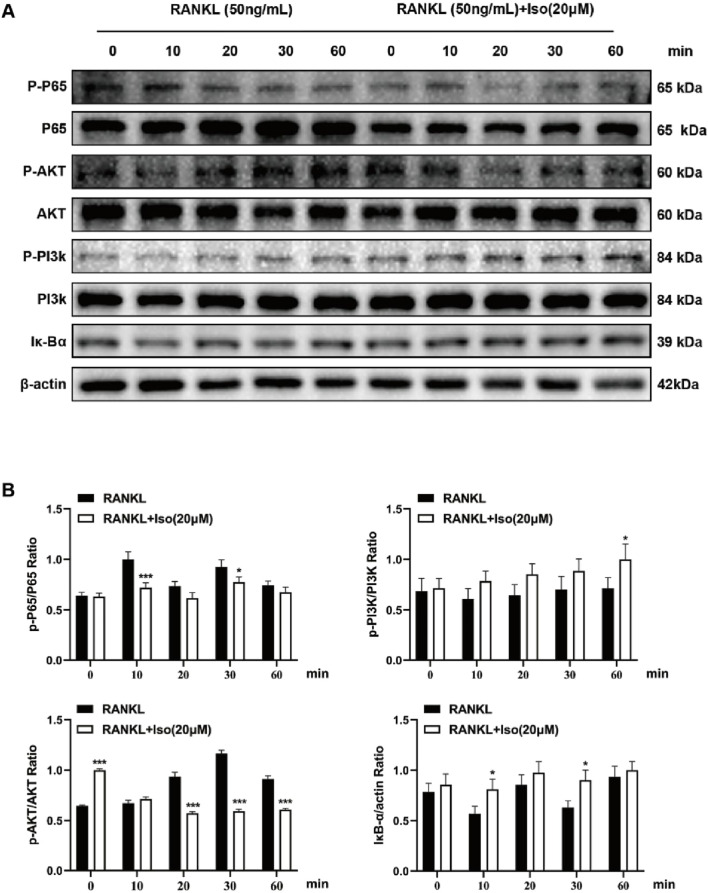
Iso modulates osteoclastogenesis via the NF-κB/PI3K-AKT signaling pathway. **(A)** Representative Western blot images demonstrating Iso-mediated inhibition of P65, AKT, PI3K phosphorylation, and IκB-α expression. **(B)** Quantitative analysis of protein levels is presented as bar graphs. Iso: Isorhamnetin. *p < 0.05, **p < 0.01, ***p < 0.001 compared to the control group (n = 3).

**FIGURE 9:**
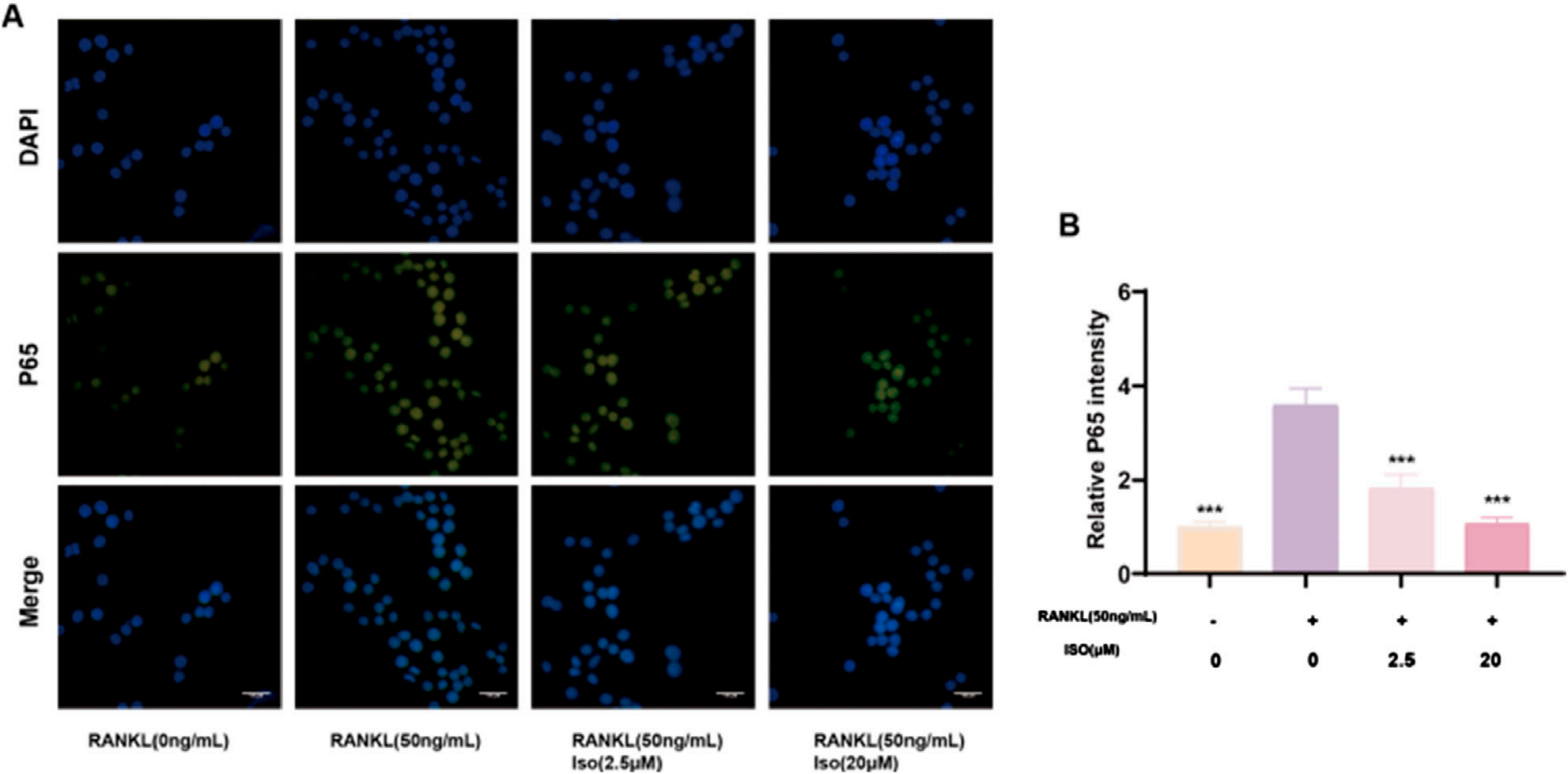
Representative fluorescence imaging reveals Iso-mediated modulation of p65 nuclear translocation. **(A)** Fluorescence mimeographs depict p65 distribution across experimental groups. **(B)** Comparative analysis of relative p65 fluorescence intensity (statistical significance indicated). Iso: Isorhamnetin. *p < 0.05, **p < 0.01, ***p < 0.001 compared to the RANKL (+) group (n = 3). Scale bars = 100 μm.

Osteoclast differentiation is regulated by various signaling pathways, including the NF-κB and PI3K-AKT cascades, a finding that aligns with the results of our Network pharmacology analysis. Western blot demonstrated that Iso (20 μM) modulated these pathways to inhibit osteoclast formation. As shown in Figures 8A,B, Iso significantly decreased p-P65 phosphorylation at 10 and 30 min. In contrast, elevated IκB-α expression was observed at both 10 and 30 min intervals (Figures 8A,B). Additionally, Iso suppressed AKT phosphorylation at 20, 30, and 60 min, while no effect on PI3K phosphorylation was observed. Furthermore, we examined NF-κB subunit P65 fluorescence levels post-Iso treatment via live-cell fluorescence microscopy. Relative to the RANKL-exposed controls, P65 signal intensity diminished significantly in Iso-treated groups, most notably at 20 μM (Figures 9A,B). Our results show that Iso suppresses phosphorylated p65 and AKT, reducing osteoclast gene transcription. This inhibition blocks osteoclast maturation. Importantly, our study reveals that Iso disrupts RANKL-driven PI3K-AKT signaling by preventing p65 nuclear translocation and transcription. It also lowers phosphorylated p65 and AKT protein levels. These findings highlight Iso’s ability to target multiple steps in osteoclast differentiation. They suggest Iso may disrupt osteoclastogenesis by weakening both NF-κB and PI3K-AKT signaling cascades.

### 3.8 Iso inhibits osteoclastogenesis via calcium signaling pathway

**FIGURE 10:**
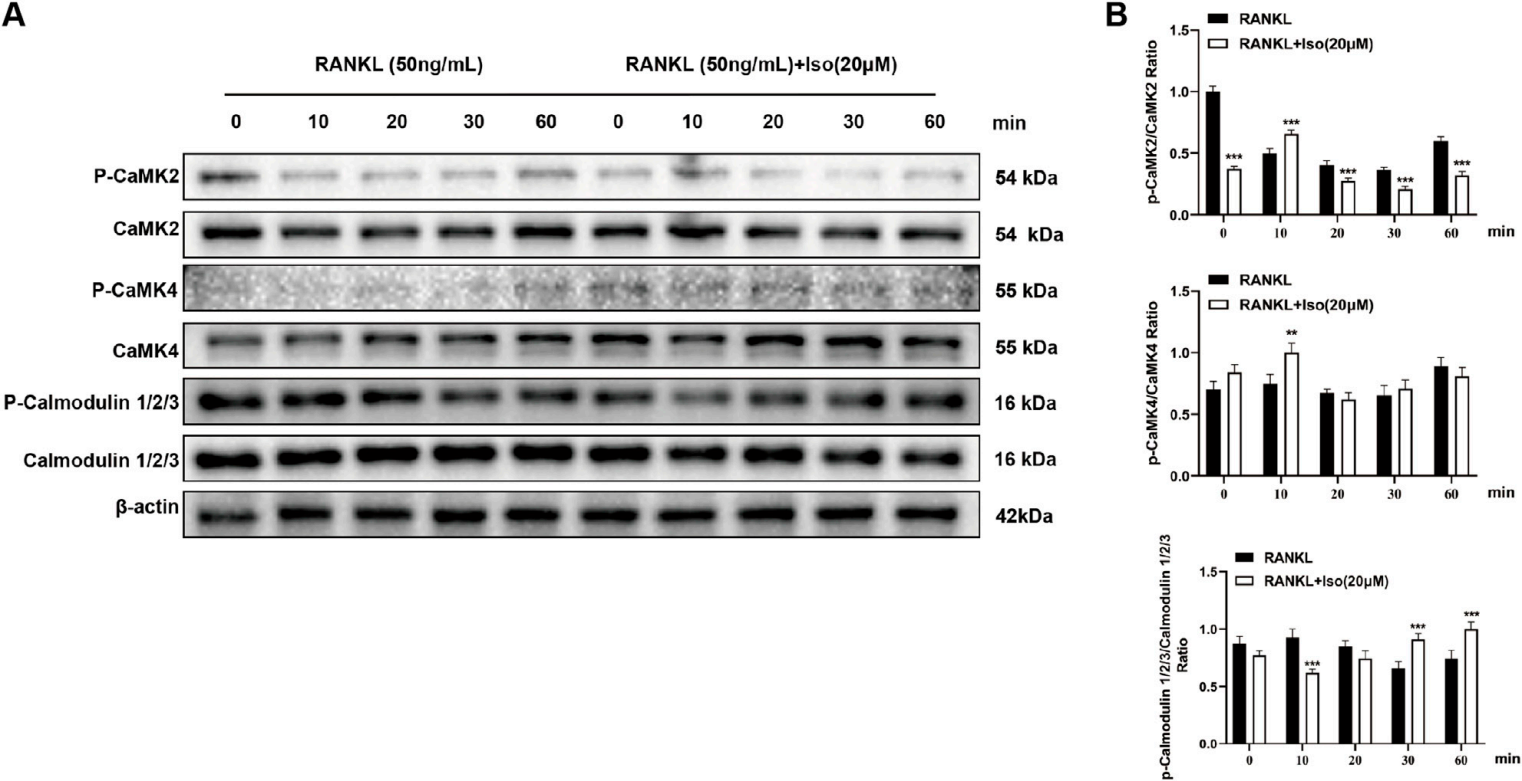
Iso suppresses the phosphorylation of calcium signaling-related proteins. **(A)** Representative Western blot images showing Iso-mediated inhibition of protein phosphorylation. **(B)** Quantitative analysis of phosphorylated protein levels is denoted as bar graphs. Iso: Isorhamnetin. *p < 0.05, **p < 0.01, ***p < 0.001 compared to the control group (n = 3).

In addition to the aforementioned signaling pathways, the interaction between RANKL and RANK can also activate the calcium ion pathway to affect osteoclasts, which is crucial for osteoclast proliferation, differentiation, gene transcription, and bone resorption. The KEGG enrichment analysis in network pharmacology also revealed this calcium ion pathway. Western blot analysis (Figures 10A,B) demonstrated a biphasic response of Iso on CaMK2 phosphorylation, with an initial increase at 10 min and a marked reduction between 20 and 60 min. A transient increase in phosphorylated CaMK4 levels was detected following Iso treatment at the 10-min interval, whereas no significant changes occurred at subsequent time points. Temporal changes in the phosphorylation of calmodulin isoforms 1/2/3 were noted, with suppression at 10 min and an increase in phosphorylation at 30 and 60 min. Collectively, our findings elucidate Iso’s modulatory influence on the RANKL-driven calcium signaling cascade, highlighting its capacity for time-dependent regulation of calcium signaling dynamics.

### 3.9 Iso exhibited binding affinity for both RANK and RANK

**FIGURE 11:**
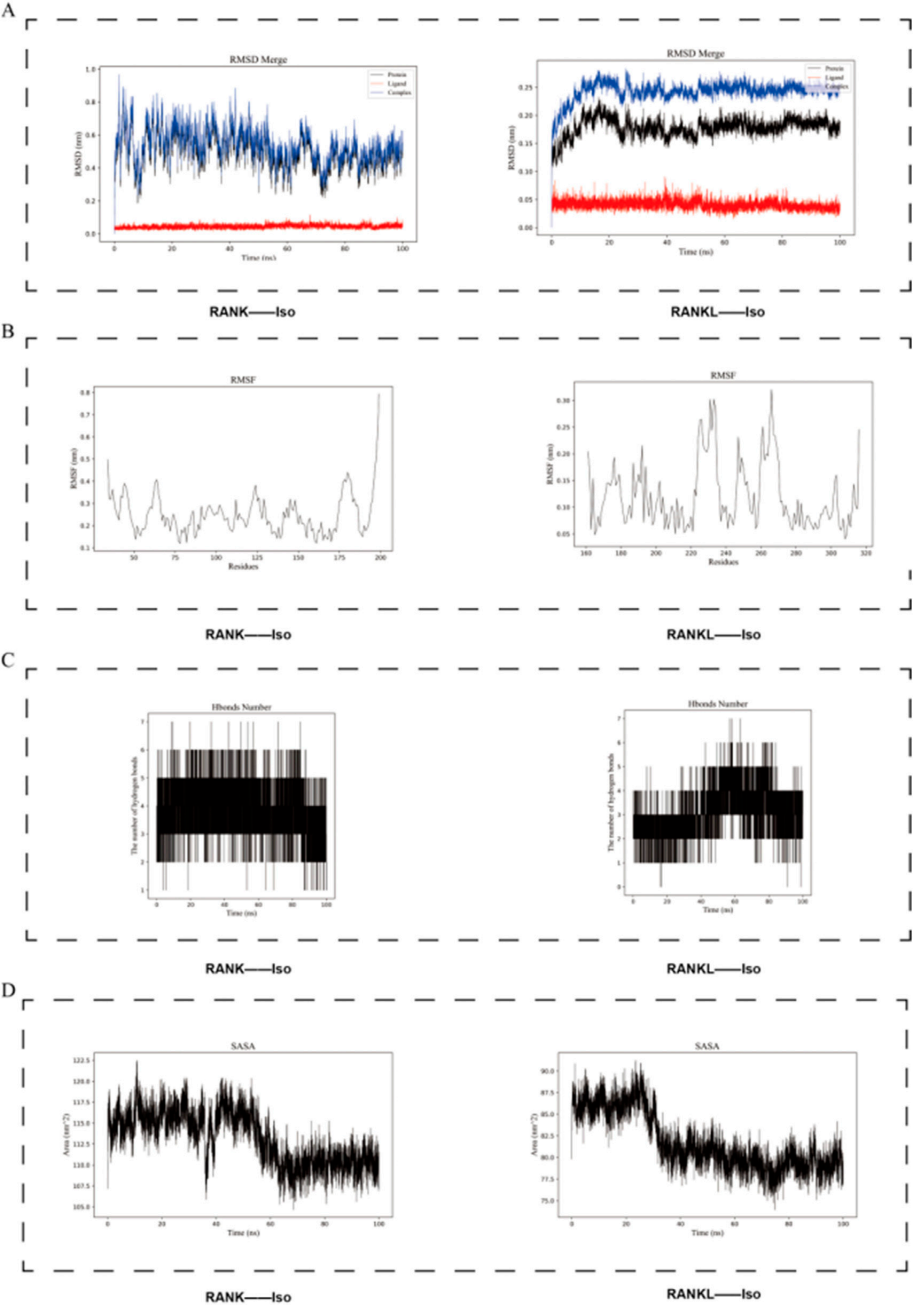
Using molecular dynamics simulations, we investigated the stability of the binding between Iso and the RANK and RANKL proteins. **(A)** The RMSD plot shows the stability of RANK protein, RANKL protein, Iso ligand, and their complexes. **(B)** RMSF determines the suitability of the interaction between Iso and RANK/RANKL proteins during the simulation time. **(C)** Hbind analysis **(D)** Solvent-accessible surface area. Iso: Isorhamnetin.

To assess protein-small molecule interactions, molecular dynamics simulations were carried out using the GROMACS software package, focusing on the specific binding of Iso to RANK and RANKL. The root-mean-square variance (RMSD), was utilized to measure structural changes in the proteins (Al-Shar’i and Al-Balas, 2019). RMSD values were computed for the RANK-Iso and RANKL-Iso complexes, generating corresponding RMSD plots. The results indicated a stable binding between the proteins and Iso. Over the initial 100 ns simulation, RANKL-Iso exhibited less fluctuation in RMSD compared to RANK-Iso (Figure 11A), suggesting a more stable interaction between RANKL and Iso. From 20 to 100 ns, RMSD values for both complexes remained within narrow ranges, implying that the protein-Iso interaction had reached equilibrium (Figure 11A).

Root-mean-square fluctuation (RMSF) analysis was conducted to evaluate the proteins’ dynamics. RMSF values for the protein-Iso complexes demonstrated lower fluctuations in the binding regions and higher fluctuations in the non-binding regions. The Iso-RANK complex generally showed greater RMSF values (nm) than the Iso-RANKL complex, indicating distinct effects of Iso binding on protein stability (Figure 11B).

Hbind analysis was carried out to assess the interactions between the proteins and the small molecule. As illustrated in Figure 11C, both RANK and RANKL formed several Hbind interactions with Iso, predominantly involving crucial residues and functional groups within the proteins.

**FIGURE 12:**
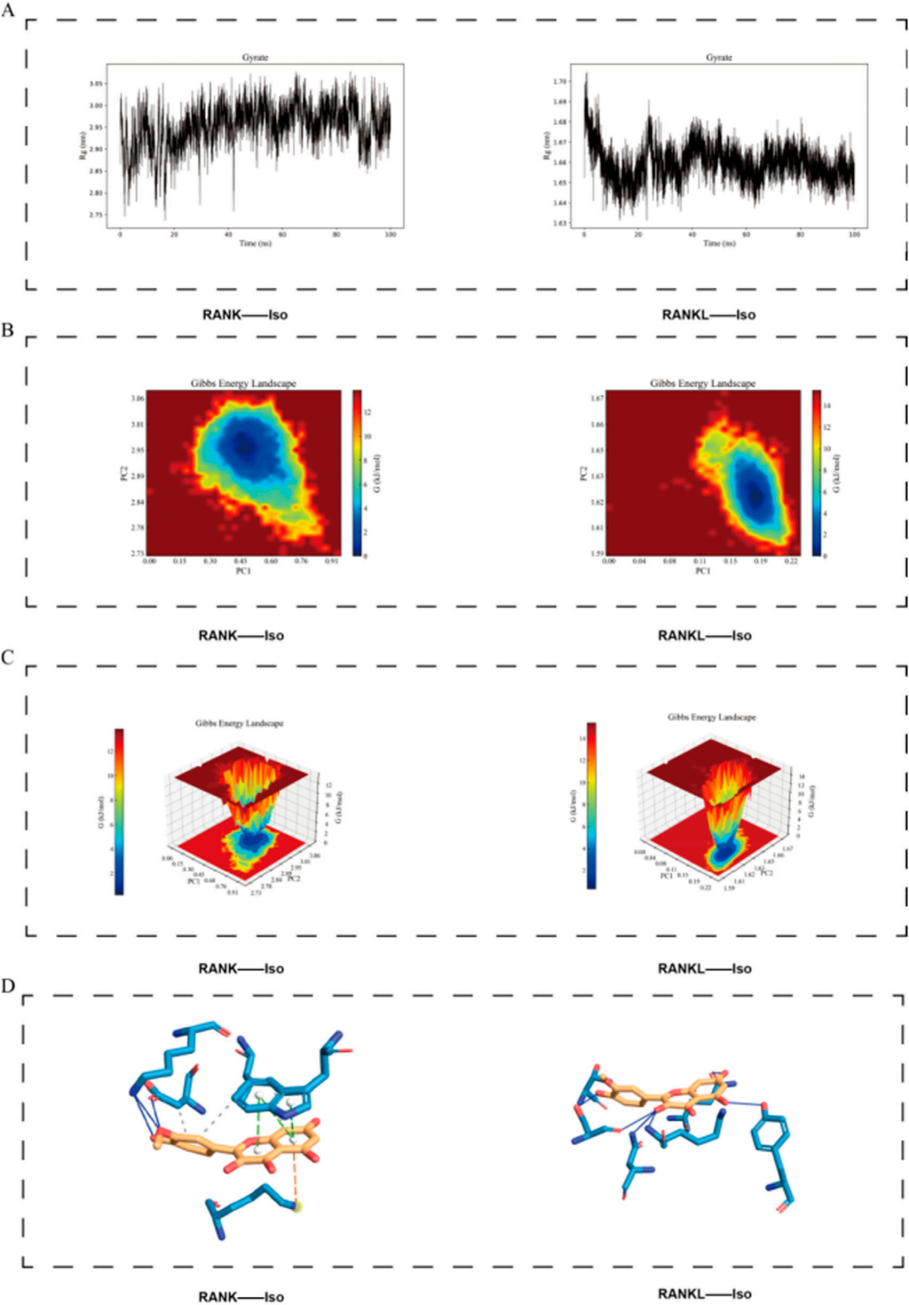
Visualization of the Stability of Iso Binding to RANK and RANKL Proteins. **(A)** RMSF determines the suitability of the interaction between Iso and RANK/RANKL proteins during the simulation time. **(B,C)** The two-dimensional and three-dimensional representations of the free energy landscape of the RANK/RANKL-Iso complex. **(D)** Representative structure of the most stable RANK/RANKL-Iso conformation extracted from the FEL analysis. Iso: Isorhamnetin.

Solvent-accessible surface area (SASA) and Radius of gyration (Rg) were employed to evaluate protein surface area and overall compactness, respectively. The results revealed that both RANK and RANKL exhibited relatively high SASA values prior to Iso binding, which decreased upon the formation of the complex, indicating a reduction in solvent exposure of the protein surfaces (Figure 11D). Figure 12A showed that Rg values for the RANK-Iso complex were higher than those for RANK alone, suggesting that Iso binding led to a more relaxed protein conformation. In contrast, the binding of Iso to RANKL resulted in lower Rg values, implying the adoption of a more compact conformation (Figure 12A).

The free energy landscape (FEL) analysis, represented through color gradients and contours, offers insights into molecular interactions, conformations, and stability within the system. Conformations that are thermodynamically favorable correspond to regions of lower free energy, whereas less stable conformations are associated with regions of higher energy. Local minima, or low-energy regions, generally represent energetically stable conformations.

The analysis of the Iso-RANK complex revealed a prominent low-energy region within the FEL, suggesting a prolonged occupation of this conformational state throughout the simulation. The molecular structure displayed relatively stable conformations primarily observed between 60 and 90 ns (Figures 12B,C). In contrast, the analysis of the Iso-RANKL complex revealed more stable protein-small molecule interactions, with the FEL exhibiting broader low-energy regions (blue areas), suggesting enhanced complex stability. Major stable conformations were observed within the 10–90 ns simulation window (Figures 12B,C). Representative structure of the most stable RANK/RANKL-Iso conformation extracted from the FEL analysis is shown in Figure 12D

In conclusion, Iso exhibited a strong binding affinity for both RANK and RANKL, aligning with the results presented in Table 3.

**TABLE 3:** Energy between Iso and protein.

Energy (KCal/Mol)	∆VDWAALS	∆E_EL_	∆E_GB_	∆E_SURF_	∆G_GAS_ ^a^	∆G_SOLV_ ^b^	∆TOTAL^c^ ^,^ ^d^
Iso/RANK	−23.33	−41.54	41.23	−3.31	−64.87	37.92	−26.95
Iso/RANKL	−33.44	−33.12	37.11	−3.91	−66.56	33.20	−33.36

^a^ ∆G_GAS_ = ∆VDWAALS + ∆E_EL_.

^b^ ∆G_SOLV_ = ∆E_GB_ + ∆E_SURF_.

^c^ TOTAL = ∆GGAS + ∆GSOLV.

^d^ TOTAL = ΔG_MMGBSA_.

∆VDWAALS, van der waals energy; ∆E_EL_, electrostatic energy; ∆E_GB_, polar solvation energy; ∆E_SURF_, Non-polar solvation energy; ∆G_GAS_, molecular mechanics term energy; ∆G_SOLV_, solvation energy; ∆TOTAL, comprehensive total energy.

### 3.10 Iso is directly integrated with RANK/RANKL

**FIGURE 13:**
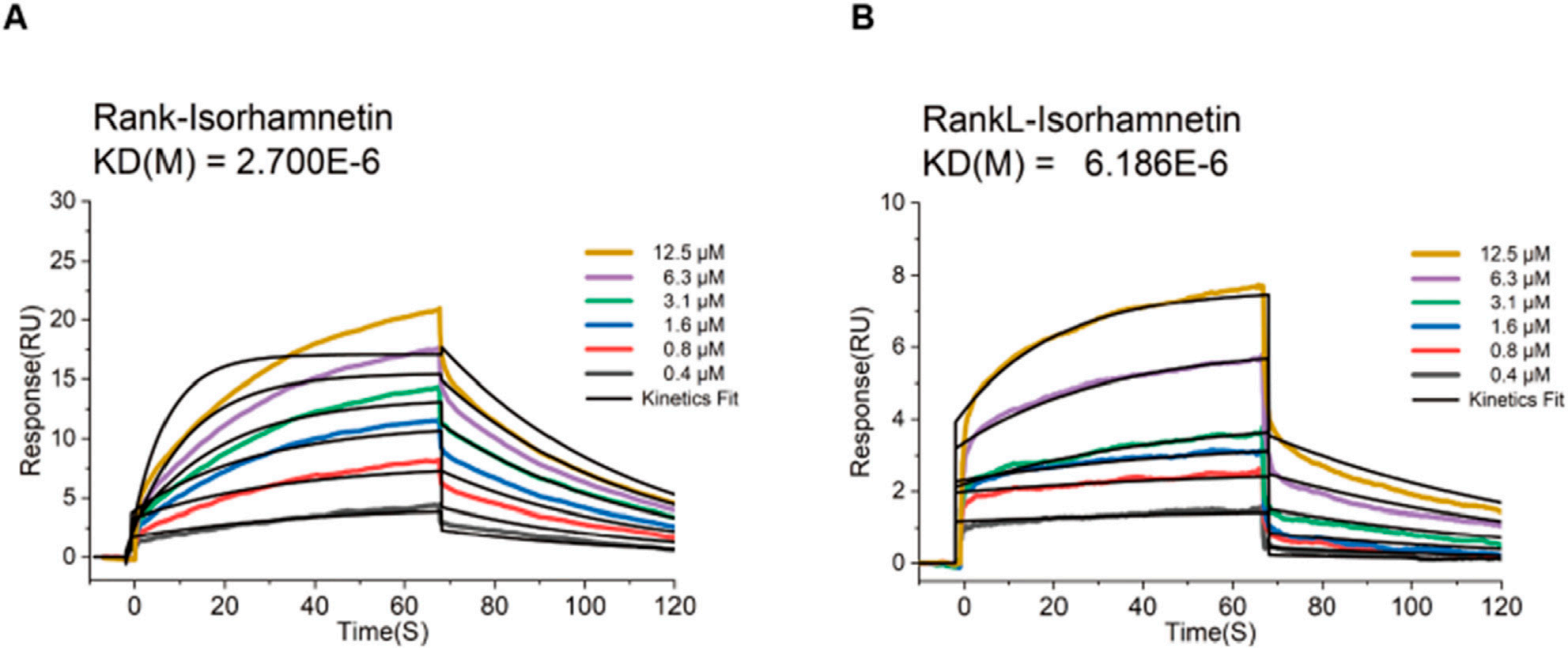
Iso blocks RANKL-RANK interaction. **(A)** Real-time binding kinetics between Iso and RANK were analyzed via surface plasmon resonance (SPR) using the Biacore T200 system. Binding responses escalated proportionally with rising Iso concentrations, confirming a concentration-dependent interaction. **(B)** Sensorgram data depicting Iso-RANKL interactions revealed a positive correlation between binding levels and Iso concentration gradients. Concurrently, Iso-RANK binding intensity exhibited a dose-dependent enhancement, further supporting its role in disrupting RANKL-RANK complex formation. KD(M), koff/kon. Iso, Isorhamnetin.

Prior molecular dynamics simulations revealed robust binding affinity of Iso for both RANK and RANKL. To validate these findings, we employed surface plasmon resonance (SPR) assays. The results confirmed that Iso binds to RANK concentration-dependent, with stronger interactions observed at higher concentrations (Figure 13A). Similarly, SPR analysis demonstrated a positive correlation between Iso-RANKL binding levels and increasing Iso concentrations, reflecting a clear gradient effect (Figure 13B). These data suggest that Iso disrupts RANKL-RANK binding, suppressing downstream pathway activation and impeding osteoclast differentiation (Figures 13A,B). Collectively, inhibition of the RANKL-RANK interaction emerges as a key mechanism through which Iso attenuates osteoclast differentiation.

### 3.11 Iso mitigates bone loss and ameliorates osteoporosis (OP)

**FIGURE 14:**
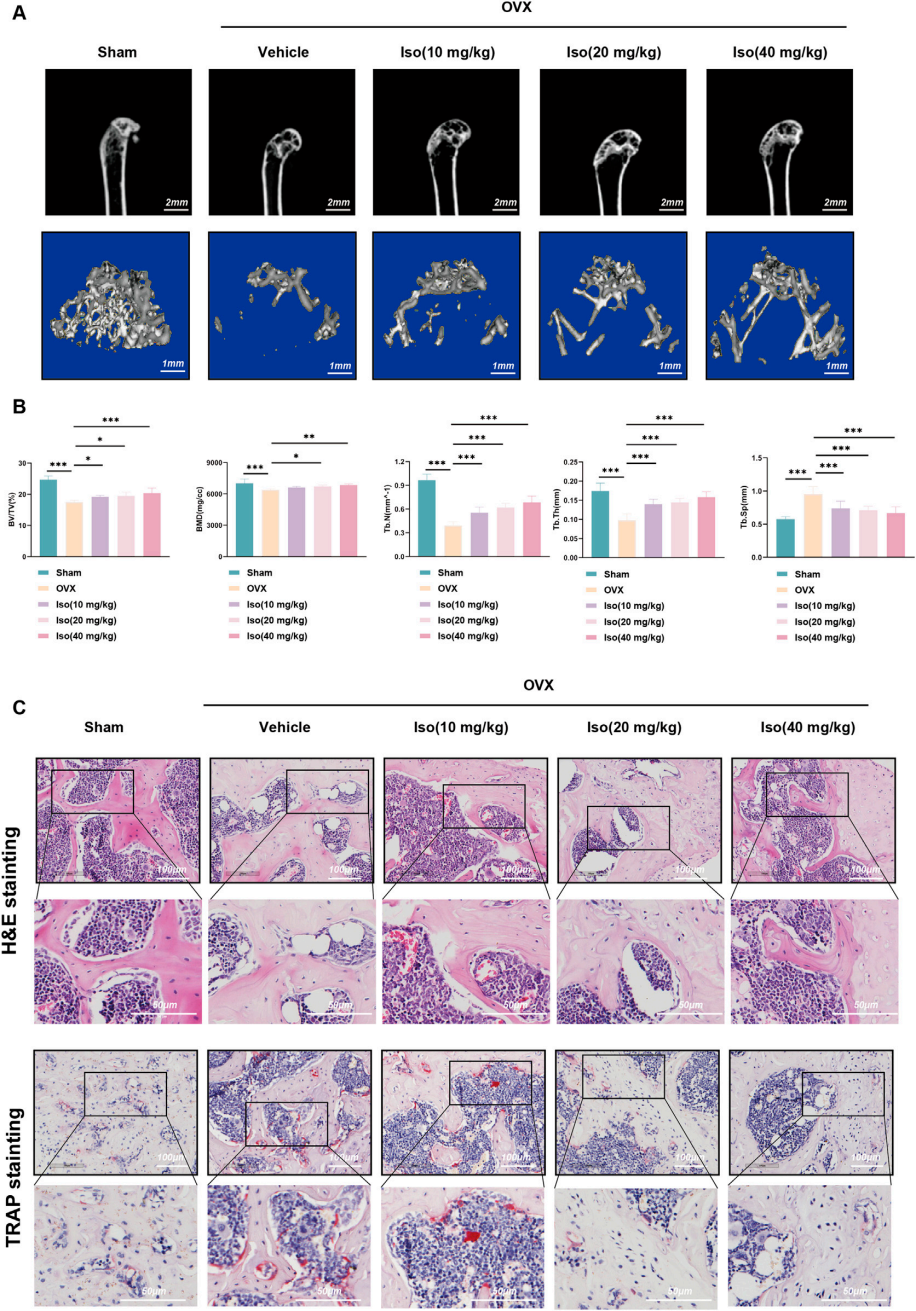
Amelioration of OVX-induced bone loss by Iso. **(A)** Representative two-dimensional and three-dimensional micro-computed tomography images across experimental groups. **(B)** Quantitative assessment of subchondral bone microarchitecture in the distal femur included measurements of bone mineral density (BMD), bone volume fraction (BV/TV), trabecular number (Tb.N), trabecular thickness (Tb.Th), and trabecular spacing (Tb.Sp). **(C)** Representative H&E and TRAP staining results of tibial plateau sections. Iso: Isorhamnetin. OVX: Ovariectomized. *p < 0.05, **p < 0.01, ***p < 0.001 compared to the Sham group (n = 6). Scale bar = 100 or 50 µm.

To confirm our *in vitro* observations and further investigate the therapeutic potential of Iso, an ovariectomy (OVX) mouse model of OP was established. Following the successful induction of the model, varying concentrations of Iso were administered intraperitoneally to the osteoporotic mice. After 8 weeks of treatment, Micro-CT and histological analyses, including H&E and TRAP staining, were performed on the hind limbs of the experimental animals. As depicted in Figures 14A–C, the OVX group displayed substantial bone loss when compared to the Sham group, with significant deterioration observed across all trabecular parameters. In contrast, Iso treatment effectively alleviated bone loss, leading to marked bone density and trabecular structure improvements. These findings suggest that Iso inhibits osteoclast-mediated bone resorption, underscoring its potential as a therapeutic approach for OP.

### 3.12 Iso suppressed expression of osteoclast-specific genes and serum bone metabolism indicators

**FIGURE 15:**
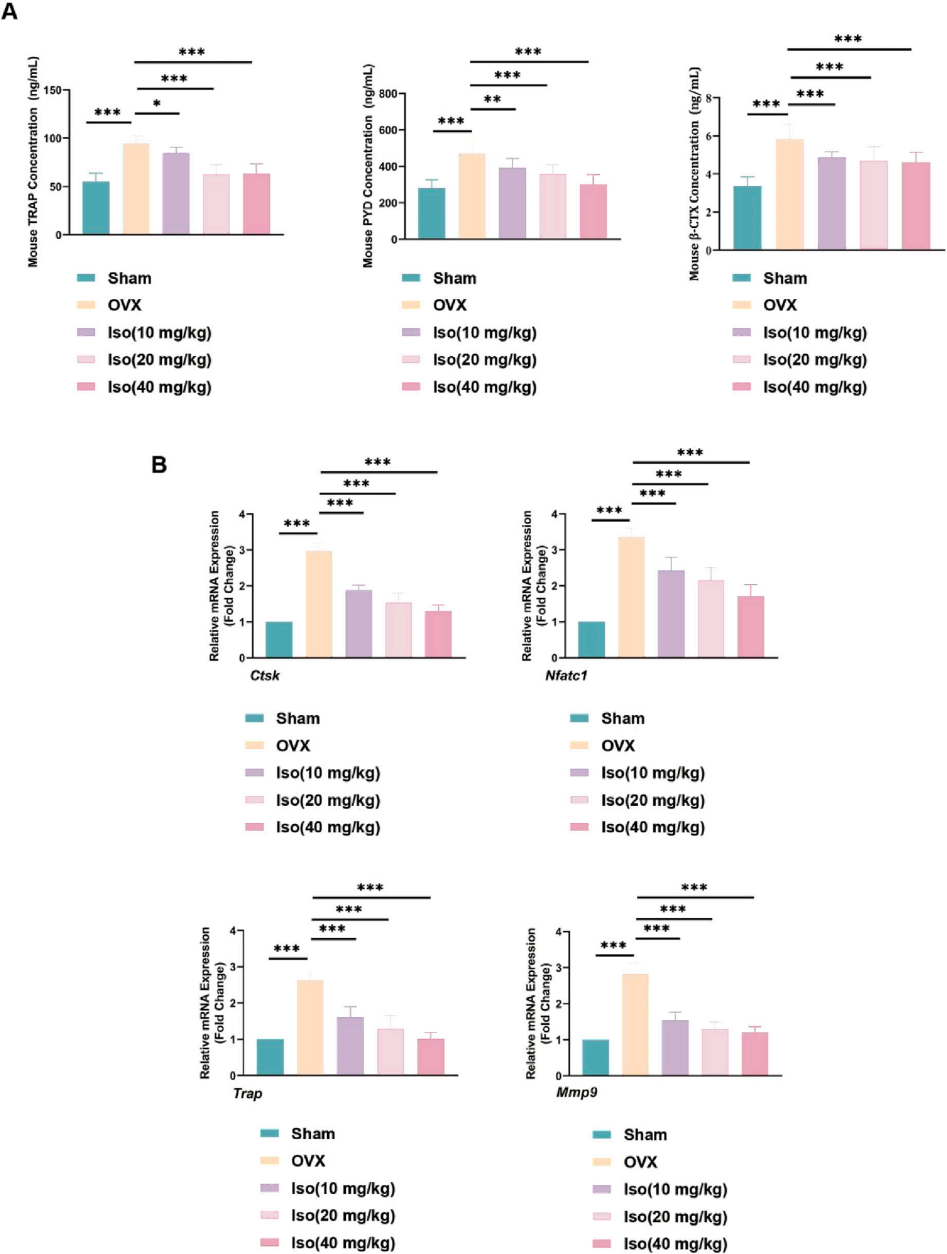
Iso suppressed expression of OC-specific genes and serum bone metabolism indicators in OVX mice. **(A)** Serum bone metabolism level. **(B)** *Nfatc1, Trap, Ctsk, and Mmp9* mRNA levels. Iso: Isorhamnetin. OVX: Ovariectomized. *p < 0.05, **p < 0.01, ***p < 0.001 compared to the OVX group (n = 6).

To further examine the specific impact of Iso on bone metabolism in the ovariectomy (OVX) mouse model, serum and bone tissue samples were collected from each group of mice, and ELISA and RT-qPCR assays were subsequently performed. These assays enabled the quantification of serum bone metabolism markers and the evaluation of gene expression associated with osteoclast function in bone tissues. As illustrated in Figure 15A, after 8 weeks of treatment, serum levels of bone resorption markers (TRAP, PYD, β-CTX) were markedly elevated in the OVX group compared to the Sham group. However, these markers displayed a decreasing trend following Iso administration, with the most notable reduction observed at the higher Iso dose (40 mg/kg). Moreover, as demonstrated in Figure 15B, RT-qPCR analysis confirmed the inhibitory effect of Iso on osteoclastogenesis, revealing a dose-dependent downregulation of osteoclast-specific genes, including *Nfatc1, Trap, Ctsk, and Mmp9*. Consistent with our *in vitro* findings, bone tissue from OVX mice exhibited upregulated expression of these genes. In contrast, Iso treatment reduced their transcriptional activity, with the extent of reduction increasing proportionally to the Iso concentration. Collectively, our data reveal that Iso exerts a suppressive action on critical osteoclast-associated proteins and exhibits bone-preserving properties against osteoporosis triggered by post-ovariectomy estrogen depletion.

## 4 Discussions

The U.S. Food and Drug Administration (FDA) has approved multiple osteoporosis (OP) treatments. These medications work by targeting bone metabolism. Examples include Denosumab, Teriparatide, and bisphosphonates. Supplements like calcium and vitamin D are also part of standard treatment plans. Bisphosphonates are often the first treatment option for patients. However, they carry risks of side effects. These include atypical bone fractures. Another potential complication is osteonecrosis of the jaw. Some studies also link bisphosphonates to atrial fibrillation. Calcium and vitamin D supplements support bone health. They are generally recommended alongside prescription therapies. Patients should discuss risks and benefits with their healthcare providers (Yuan et al., 2019). Denosumab is a monoclonal antibody used to treat OP. It reduces bone loss by blocking receptor activator for nuclear factor-κB ligand (RANKL), a protein involved in bone breakdown. Stopping Denosumab treatment can trigger a “rebound effect”. This rebound effect involves a sudden rise in bone turnover markers and heightened osteoclast (OC) activity (Kim et al., 2024). Such rapid changes may accelerate bone mineral density (BMD) loss. They also increase the likelihood of multiple spinal fractures (Grassi et al., 2021). Clinicians must monitor patients carefully after discontinuing Denosumab. As an anabolic bone therapeutic, teriparatide improves bone mineral density and microarchitecture by promoting osteoblast differentiation and bone formation, thereby lowering fracture susceptibility. Clinical evidence indicates its significant efficacy in OP management, particularly among high-risk patients, where it effectively elevates bone density and reduces fracture rates (Blick et al., 2009). Additionally, teriparatide has been employed in addressing atypical femoral fractures linked to prolonged bisphosphonate use, with studies reporting accelerated fracture repair and enhanced bone quality (Chiang et al., 2013; Gao et al., 2022). Nevertheless, teriparatide therapy is associated with adverse effects, notably a potential elevation in osteosarcoma risk at elevated doses. This association is primarily derived from preclinical models demonstrating osteosarcoma incidence following high-dose teriparatide exposure. Consequently, clinical administration is limited to duration and dosage-restricted regimens to minimize safety concerns (Karatoprak et al., 2012).

Romosozumab, a groundbreaking monoclonal antibody targeting sclerostin, enhances bone formation while suppressing resorption, substantially elevating bone mineral density and lowering fracture incidence in OP management (Fontalis et al., 2019; Bovijn et al., 2020). However, clinical studies have reported potential cardiovascular adverse events, such as myocardial infarction and coronary revascularization, linked to its administration (Bovijn et al., 2020). Additionally, mild hypersensitivity responses occur in approximately 4.1% of patients, necessitating vigilant clinical monitoring (Chavassieux et al., 2019). Therefore, a cautious evaluation of Romosozumab’s therapeutic advantages relative to its cardiovascular and hypersensitivity profiles is essential in clinical practice (Chavassieux et al., 2019; Bovijn et al., 2020). The ideal management approach for OP requires dual modulation of OC activity and osteoblast activation. Contemporary treatments primarily target osteoclastic bone resorption, which may inadvertently induce complications like adynamic bone pathology following prolonged anti-resorptive therapy (Recker and Armas, 2011). This condition stems from excessive inhibition of bone remodeling mechanisms, potentially degrading skeletal integrity and biomechanical competence.

Despite expanding therapeutic possibilities, current pharmacological interventions for OP remain limited by inadequate long-term safety profiles. In elderly patients with comorbidities, cumulative adverse effects frequently necessitate treatment cessation, highlighting the need for safer alternatives. This challenge has revitalized exploration of phytochemicals exhibiting dual osteoanabolic and anti-resorptive properties. Among these, flavonoids have gained prominence for their multifaceted benefits in bone health, including cancer risk reduction and minimal gastrointestinal toxicity (Diao et al., 2024). Research indicates that flavonoids enhance bone mineral density by balancing remodeling processes: suppressing excessive resorption while fostering osteoblast-osteoclast equilibrium to preserve skeletal homeostasis. Their bone-protective effects are mediated through antioxidant and anti-inflammatory mechanisms, alongside regulation of signaling pathways governing osteogenesis and osteoclastogenesis (Sekaran et al., 2022; Scarpa et al., 2024). Unlike monoclonal antibodies with singular targets, flavonoids exert multi-dimensional modulation of bone metabolism, including epigenetic regulation. For example, quercetin, a phytoestrogenic flavonoid, activates the BMP-2/Smad1/4/RUNX2/OSX/OPN cascade to stimulate bone marrow stromal cell proliferation and osteogenic differentiation, while amplifying Smad1 phosphorylation (Pang et al., 2018). Concurrently, quercetin suppresses RANKL expression, a master regulator of osteoclastogenesis, thereby attenuating pathological bone loss (Kim et al., 2019). Additionally, natural compounds typically induce fewer side effects during treatment, particularly when contrasted with traditional pharmaceutical therapies, as they are less likely to cause gastrointestinal discomfort (Chen et al., 2023).

Iso is a flavonoid found in medicinal plants like *Hippophae rhamnoides L. (H. rhamnoides L.)* and Ginkgo biloba (Ginkgo biloba L.). It also occurs in dietary sources such as onions and apples. Studies show it has low toxicity and therapeutic benefits, including antioxidant and anti-inflammatory effects (Gomez-Zorita et al., 2023). SwissADME computational tools predict Isorhamnetin meets key drug development criteria. These include good bioavailability, compliance with Lipinski’s rules, no structural safety alerts (PAINS/Brenk), and ease of synthesis (Seukep et al., 2025). ProTox-II toxicity assessments estimate its LD50 at 5000 mg/kg. This classifies it under Category V, indicating low acute toxicity and minimal risk. *In vivo* studies use doses like 100 mg/kg, well below this safety limit, confirming its tolerability (Gao et al., 2017). Its pharmacokinetic and pharmacological features support its therapeutic potential. To improve flavonoid bioavailability, researchers developed a standardized complex called IPHRFPPEF-PC. This formulation enriches Isorhamnetin content. It boosts plasma concentrations by 1.99 fold and maintains a safe profile, addressing bioavailability limitations (Gore et al., 2025).

The research demonstrated that Iso inhibits RANKL-induced reactive oxygen species (ROS) generation, thereby modulating OC differentiation pathways through ROS-dependent mechanisms (Zhou et al., 2019). The inhibition of the RANK-RANKL signaling pathway, which serves a pivotal function in OC biology, effectively obstructs the differentiation of mature OCs and their associated bone resorptive activity, thus mitigating bone loss. Consequently, this pathway is frequently targeted in the development of OP therapies, as demonstrated by the use of Denosumab. First, we tested our hypothesis by investigating Iso mechanism of action. We conducted molecular dynamics simulations to predict its binding to the RANKL/RANK complex. Experimental validation followed using surface plasmon resonance (SPR) technology. These methods are well-established for analyzing receptor-protein interactions with natural compounds (Forssén et al., 2018; Al-Shar’i and Al-Balas, 2019). Our findings show that Iso competitively blocks RANKL/RANK binding. This inhibition effectively suppresses OC formation.

We conducted *in vitro* experiments to assess Iso’s effects on OC differentiation and activity. The results showed that Iso strongly inhibits OC differentiation in a dose-dependent manner. Additionally, Iso reduced the expression of key OC-related genes and proteins, such as NFATc1, CTSK, MMP9, and c-Fos. It also blocked RANKL-induced ROS production, which drives OC formation. These findings indicate that Iso effectively inhibits osteoclastogenesis. To better understand its action, we further studied Iso’s molecular mechanisms in OC maturation and differentiation.

The activation and regulation of NFATc1 are essential for the proper formation and function of OCs, as it controls the expression of genes necessary for OC differentiation and activity (Jianwei et al., 2022). The role of NFATc1 in osteoclastogenesis is underscored by its regulation through various signaling pathways. For instance, RANKL signaling pathway is a primary driver of NFATc1 activation (Jung et al., 2019). As a key regulator of osteoclastogenesis, the absence of NFATc1 disrupts the normal differentiation of OCs (Charles et al., 2012). Our *in vitro* and *in vivo* data show that Iso treatment significantly reduces NFATc1 gene and protein expression. It also lowers NFATc1 activity.

Our network pharmacology KEGG enrichment analysis highlights three key pathways: the MAPK, PI3K-AKT, and calcium ion signaling pathways. These pathways are strongly linked to OC formation, as shown in prior studies. The research demonstrated reported that Iso suppresses RANKL-activated MAPK/NF-κB/AKT signaling (Zhou et al., 2019). However, their work focused only on the late stage of OC differentiation. Early-stage mechanisms, such as initiation and intermediate phases, remain understudied in current research. Comprehensive investigations into underlying mechanisms spanning the entire differentiation timeline remain imperative to fully elucidate Iso’s regulatory effects.

The Mitogen-Activated Protein Kinases (MAPK) pathway is a crucial signaling cascade that regulates osteoclastogenesis and bone resorptiont (Mun et al., 2020). The MAPK family encompasses several key kinases, including P38, JNK, and ERK, which are integral to cellular processes such as proliferation, differentiation, stress responses, and apoptosis (Park et al., 2019; Li et al., 2021). P38 is particularly implicated in regulating OC maturation through activating the downstream transcription factor MITF. This cascade modulates the expression of OC-specific genes, such as TRAP and CTSK (Lee K. et al., 2018). JNK is a critical transcription factor for maintaining OC bone resorption function. In the absence of JNK, bone resorption is markedly impaired, even in the presence of RANKL. Studies indicate that the positive regulation of JNK counteracts the apoptotic effects induced by RANKL in OCs. The multifaceted influence of JNK on OC function, survival, and activity underscores its central role in the intricate signaling network that governs bone homeostasis (Ikeda et al., 2008; Huang et al., 2022). We found that RANKL stimulation significantly increased JNK phosphorylation in RAW 264.7 cells. However, Iso treatment inhibited MAPK pathway activity. Specifically, Iso reduced phosphorylation of JNK and P38. It had no notable impact on ERK phosphorylation.

The PI3K/Akt pathway is critical for cellular signaling, especially in controlling OC activity (Lu et al., 2024). PI3K (phosphatidylinositol 3-kinase) regulates key processes like cell growth, survival, and intracellular transport (Shinohara et al., 2012). Following RANKL stimulation, PI3K activation triggers phosphorylation of signaling proteins such as Akt (Zheng et al., 2024). Akt, a serine/threonine kinase, drives OC formation by phosphorylating downstream targets like NFATc1 (Wang et al., 2023). Blocking Akt phosphorylation reduces OC differentiation and bone resorption activity (Qu et al., 2014). The PI3K/Akt pathway also interacts with NF-κB signaling. Together, these cascades coordinate cellular functions (Liu et al., 2022). NF-κB signaling is widely recognized as central to OC formation and activation (Tian et al., 2022). Upon stimulation by external factors, IκBα undergoes ubiquitination, making it prone to recognition and degradation by the ubiquitin-proteasome system. This sequence of events ultimately leads to the proteolytic breakdown of IκBα (Mathes et al., 2008). Experimental data indicate that p65, an NF-κB subunit, promotes proapoptotic gene expression in OCs. Conversely, a reduction in osteoclastogenesis is observed when the activity of p65 is diminished (Yang et al., 2021). Studying these signaling pathways advances our understanding of OC biology. It also highlights new therapeutic targets for bone disorders. Notably, Iso treatment suppressed key proteins in both the PI3K/Akt and NF-κB pathways across multiple time points. We used RANKL-induced OC differentiation and Western blotting to analyze these effects. Iso strongly inhibited phosphorylated Akt in the PI3K/Akt pathway at 20, 30, and 60 min. However, phosphorylated PI3K levels rose at 60 min. In the NF-κB pathway, Iso reduced p65 phosphorylation at 10 and 30 min. It also blocked IκBα degradation at these time points. This reduction lowered c-Fos and NFATc1 protein levels, ultimately curbing OC differentiation. These findings suggest that Iso combats OC formation by suppressing RANKL-driven PI3K/Akt and NF-κB signaling.

Beyond the pathways discussed earlier, the RANKL-RANK interaction activates calcium signaling to control OC activity (Park et al., 2017). During early OC differentiation, RANKL binding activates TRAF6. This stimulates PLCγ to produce IP3, which triggers calcium release from the endoplasmic reticulum (Lee J.-U. et al., 2018). Rising calcium levels activate calcineurin via calmodulin (CaM). Calcineurin dephosphorylates NFAT, exposing its nuclear localization signals. This allows NFAT to enter the nucleus and drive NFATc1 gene expression (Ren R. et al., 2021). Western blot data showed that Iso most strongly inhibits CaMK2 phosphorylation at 20, 30, and 60 min. However, CaMK4 phosphorylation unexpectedly increased at 10 min. Phosphorylation of calmodulin isoforms 1/2/3 decreased at 10 min but rose at 30 and 60 min. These mixed results may reflect crosstalk between calcium signaling and other pathways. Together, Iso likely disrupts OC formation by altering RANKL-induced calcium signaling dynamics.

ROS are essential for OC formation and activity. Research shows ROS regulate OC differentiation and function by influencing cellular signaling pathways. Bone homeostasis depends on balanced ROS levels to avoid excessive bone loss or impaired bone formation (Schröder, 2019). During osteoclastogenesis, RANKL activates NADPH oxidase 1 (Nox1). Nox1-produced ROS then trigger pathways like MAPK and NF-κB. These pathways drive NFATc1 and c-Fos expression, which control OC differentiation (An et al., 2019). Our data reveal that Iso reduces intracellular ROS levels during osteoclastogenesis. This suggests ROS suppression contributes to Iso’s anti-osteoclastogenic mechanism.

To further explore the *in vivo* effects of Iso on OP, an OVX model was employed. A comprehensive analysis of both bone and blood samples indicated that intraperitoneal injection of Iso markedly reduced bone loss in OVX mice. Furthermore, in the OVX+Iso group, the expression of key OC-specific genes in bone tissue was substantially downregulated compared to the OVX group, suggesting that Iso effectively modulates OC activity at the transcriptional level.

Our study demonstrates that Iso inhibits OC differentiation through two key mechanisms. First, it suppresses ROS generation. Second, it reduces the expression of critical genes and proteins like c-Fos, MMP9, CTSK, and NFATc1. These actions disrupt RANK-RANKL interactions and modulate signaling pathways, including MAPK, PI3K/Akt, NF-κB, and calcium cascades. These findings position Iso as a promising candidate for OP treatment. By blocking excessive bone resorption, Iso could help maintain bone density and lower fracture risks. This is particularly relevant for postmenopausal women and high-risk groups. For patients unable to tolerate current OP drugs or those with contraindications for standard therapies, Iso offers a potential alternative. Its safety profile, demonstrated in preclinical studies, and ability to slow bone loss provide new options for tailored clinical management.

Our study has several limitations. First, we focused only on the MAPK, PI3K/Akt, NF-κB, and calcium signaling pathways. Iso’s effects on other OC-related pathways or signaling crosstalk remain unexamined. Second, we did not test Iso in OC-osteoblast co-culture systems. Such models could better replicate *in vivo* bone remodeling dynamics. Third, all experiments used murine-derived cells. Human cell studies are needed to confirm Iso’s clinical relevance for OP treatment.

## 5 Conclusion

**FIGURE 16:**
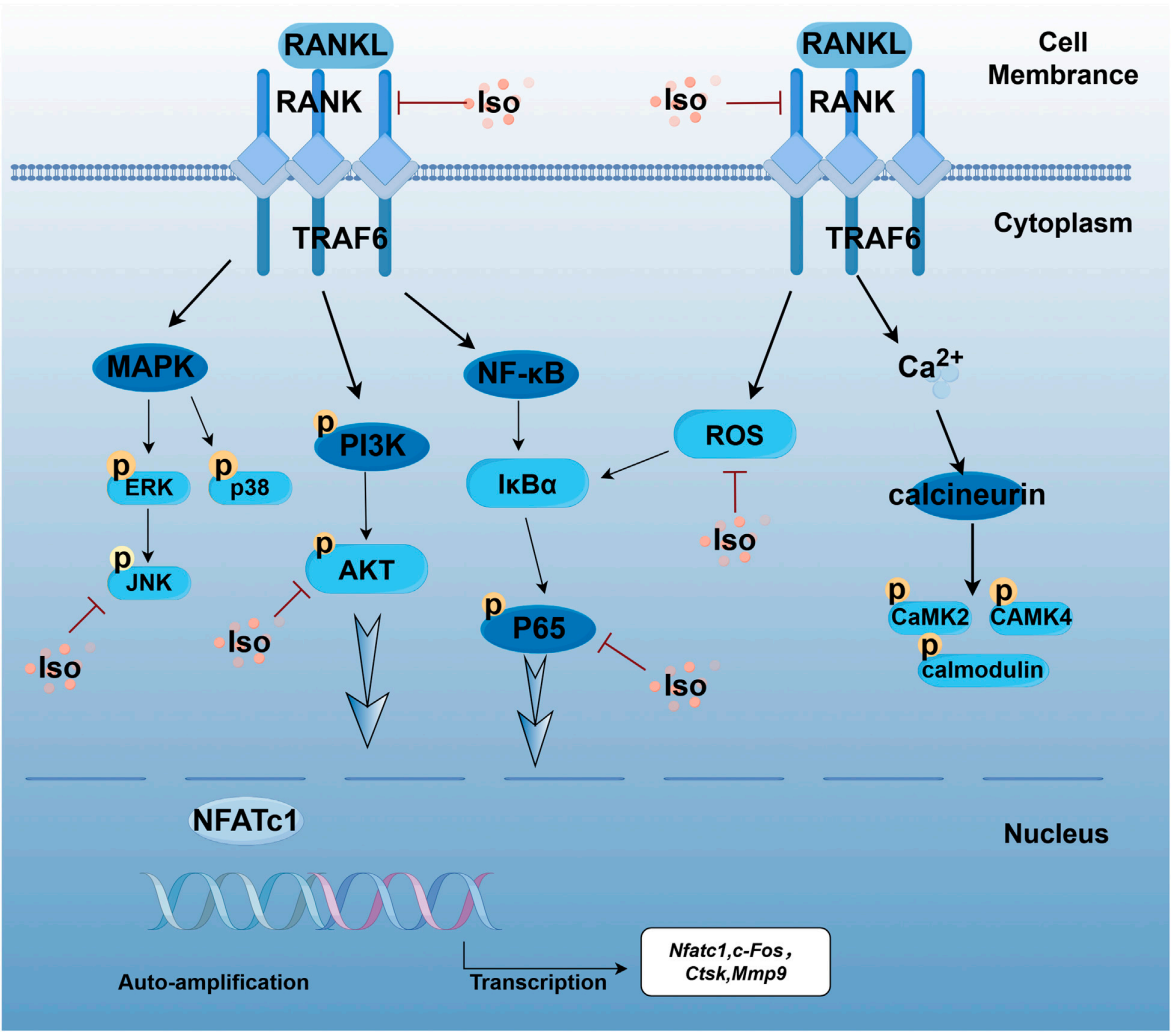
Schematic illustration of the mechanism by which Iso inhibits RANKL-induced osteoclastogenesis. Iso competes for binding with RANKL-RANK, thereby interfering with their interaction. Furthermore, Iso inhibits the phosphorylation of key signaling pathways, including MAPK, NF-κB/PI3K-AKT, and Ca^2+^ pathways, as well as calcium ion channels. This interference results in the reduced self-transcription of NFATc1 and a subsequent downregulation of OC-specific genes. Collectively, these findings suggest that Iso impedes osteoclastogenesis by blocking RANKL-RANK binding and inhibiting the transcriptional activation of NFATc1. This image was drawn using the Figdraw platform.

In conclusion, this investigation presents compelling evidence that the natural compound Iso effectively inhibits RANKL-induced osteoclastogenesis. This inhibition is mediated by suppressing ROS and RANKL-RANK binding, which downregulates the activity of key signaling pathways, including MAPK, NF-κB, and PI3K/AKT. Furthermore, the expression of NFATc1 and other OC-specific genes is markedly reduced (Figure 16). These findings underscore the potential of Iso as a promising novel anti-resorptive agent for treating osteoporosis.

## Data Availability

The original contributions presented in the study are included in the article/Supplementary Material, further inquiries can be directed to the corresponding author.
